# Objective monitoring of motor symptom severity and their progression in Parkinson’s disease using a digital gait device

**DOI:** 10.1038/s41598-025-09088-7

**Published:** 2025-07-15

**Authors:** Tamara Raschka, Jackrite To, Tom Hähnel, Stefano Sapienza, Alzhraa Ibrahim, Enrico Glaab, Heiko Gaßner, Ralph Steidl, Jürgen Winkler, Jean-Christophe Corvol, Jochen Klucken, Holger Fröhlich

**Affiliations:** 1 Department of Bioinformatics, Fraunhofer Institute for Algorithms and Scientific Computing (SCAI), Schloss Birlinghoven, 53757 Sankt Augustin, Germany; 2 Bonn-Aachen International Center for IT, University of Bonn, Friedrich Hirzebruch-Allee 6, 53115 Bonn, Germany; 3 Department of Neurology, University Hospital and Faculty of Medicine Carl Gustav Carus, TUD Dresden University of Technology, Dresden, Germany; 4 Luxembourg Centre for Systems Biomedicine, University of Luxembourg, 4367 Esch-sur-Alzette, Luxembourg; 5 Department of Molecular Neurology, University Hospital Erlangen, Friedrich-Alexander-Universität Erlangen-Nürnberg, 91054 Erlangen, Germany; 6 Machine Learning and Data Analytics Lab, Department of Artificial Intelligence in Biomedical Engineering, Friedrich-Alexander-Universität Erlangen-Nürnberg (FAU), Erlangen, Germany; 7 Portabiles HealthCare Technologies, Erlangen, Germany; 8 Department of Neurology, Inserm, CNRS, Assistance Publique Hôpitaux de Paris, Pitié-Salpêtrière Hospital, Paris Brain Institute – ICM, Sorbonne Université, Paris, France

## Abstract

Digital technologies for monitoring motor symptoms of Parkinson’s Disease (PD) underwent a strong evolution during the past years. Although it has been shown for several devices that derived digital gait features can reliably discriminate between healthy controls and people with PD, the specific gait tasks best suited for monitoring motor symptoms and especially their progression, remain unclear. Furthermore, the potential benefit as endpoint in a clinical trial context has not been investigated so far. In this study we employed a digital gait device manufactured by Portabiles HCT, which has been used by 339 patients within the LuxPark cohort (n = 161, Luxembourg) as well as within routine clinical care visits at the University Medical Center Erlangen (n = 178, Erlangen, Germany). Linear (mixed) models were used to assess the association of task-specific digital gait features with disease progression and motor symptom severity measured by several clinical scores. Furthermore, we employed machine learning to evaluate whether digital gait assessments were prognostic for patient-level motor symptom progression. Overall, digital gait features derived from Portabiles digital gait device were found to effectively monitor motor symptoms and their longitudinal progression. At the same time the prognostic performance of digital gait features was limited. However, we could show a strong reduction in required sample size, if digital gait features were employed as surrogates for traditional endpoints in a clinical trial context. Thus, Portabiles digital gait device provides an effective way to objectively monitor motor symptoms and their progression in PD. Furthermore, the digital gait device bears strong potential as an alternative and easily assessable endpoint predictor in a clinical trial context.

**Supplementary Information:**

The online version contains supplementary material available at 10.1038/s41598-025-09088-7.

## Introduction

Gait abnormalities in people with Parkinson’s Disease (PD) are a major motor symptom, along with tremor, bradykinesia, and rigidity, and have a major impact on patients’ mobility and quality of life^1–3^. Signs of impaired gait patterns are reduced gait speed and step length, increased axial rigidity, impaired rhythmicity, and freezing of gait^2,4^. In the early stages of the disease, slower gait speed, shortened step length, and reduced arm swing during walking are the most common signs of gait abnormalities, while in the later stages, movements become even more slow, gait shuffling and freezing of gait appears and patients are thus prone to falls^4^. With progression into an advanced stage, gait abnormalities, including balance and postural control, become worse^4^. Thus, investigating patient’s gait impairment is crucial for evaluating the stage of disease progression. Furthermore, objective monitoring of gait could help defining an optimal schedule of when to treat a patient, whether to adjust the dose or whether to switch from one treatment to another.

In the past, Digital technologies for monitoring motor symptoms of PD have evolved from non-portable in-lab devices to wearable sensors and at-home phone applications^5^. While each technology has its advantages, wearable sensors are able to measure gait parameters like stride length, gait speed, or the lateral excursion of each patient quantitatively and potentially also in an outpatient setting , while being portable, light weight, less expensive, and easy to use, for patients, as well as, for clinicians^5^. However, before any application in clinical care, such technologies must be carefully assessed regarding technical robustness as well as medical diagnostic validity^6^. Importantly, a digital gait sensor can only reliably be used in routine medical care after regulatory approval, requiring proof of its safety. Besides that, trustable digital gait features require clear evidence that these sensor measurements correlate significantly with established clinical outcomes such as MDS-Unified Parkinson’s Disease Rating Scale (MDS-UPDRS)^7^. Although digital gait features have been explored in several studies in PD research, most studies solely focus on distinguishing PD patients from healthy controls in cross-sectional manner^5,8–13^. Some studies also classify patients into multiple disease stages^14–17^, but studies rarely describe the associations between PD progression and gait directly^4–6^. Furthermore, there is a lack of studies investigating the reproducibility of findings from the same gait device across several PD cohorts. Taken together, it is still unclear to date whether digital gait sensors can reproducibly and objectively monitor motor symptoms and their progression over time and whether in that regard there is a demonstrable benefit to classical clinical scales.

The ERA PerMed funded EU-wide project DIGIPD (Validating DIGItal biomarkers for better personalized treatment of Parkinson’s Disease—https://www.digipd.eu) tried to fill a gap by evaluating the potential benefit of digitally assessed PD symptoms compared to established clinical outcomes across several cohorts in Europe. In this study, we focused on a sensor-based digital gait device clipped to the shoe of a patient while walking (https://www.portabiles-hct.de/en/product/). The device is currently registered for regulatory approval at the US Food and Drug Administration (FDA) and is a certified class I medical device (MDR) with a CE mark in Europe. We investigated data recorded by the device in two independent longitudinal cohorts (one from Germany, one from Luxembourg) and while performing different gait tasks in the clinic. More specifically, we initially modelled PD progression based on several established outcome measures derived from the MDS-UPDRS by fitting a latent time joint mixed-effect model (LTJMM)^18^ to longitudinal data from both studies. This allowed us to estimate each patients’ true rate of progression and to align disease trajectories on a common disease timescale, considering the fact that patients may have progressed differently at baseline. Following that, linear (mixed) models were used to evaluate the association of digital gait features with estimated progression rates as well as UPDRS derived outcomes. In that regard we also explored, in how far a machine learning model could forecast an individual’s disease progression rate solely based on digital gait features. Finally, we investigated the potential benefit of using a digital gait predicted UPDRS III compared to the original UPDRS III as an endpoint in a clinical trial.

## Material and methods

### Cohorts

We analyzed data from two independent PD cohort studies: (1) the Luxembourg Parkinson’s Study (LuxPARK, NCT05266872)^19^ and (2) from the University Medical Center Erlangen, Germany (Erlangen). LuxPARK is an ongoing observational cohort of patients at all disease stages. Data was collected periodically up to four years of follow-up between 2015 and 2022. Data from Erlangen contained information from patients at all disease stages collected during multiple irregular clinical routine visits between 2011 and 2022. For both studies, we used multiple clinical scores as outcomes in our analysis. In LuxPARK these scores were MDS-Unified Parkinson’s Disease Rating Scale (MDS-UPDRS) I, MDS-UPDRS II, MDS-UPDRS III^7^, Tremor dominance (TD) score^20^, Postural Instability and Gait Dysfunction (PIGD) score^20^, and an axial score. The axial score was calculated by summing MDS-UPDRS III sub-items 1 (speech), 2 (facial expression), 9 (arising from chair), 10 (gait), 11 (freezing of gait), 12 (postural instability), and 13 (posture), which is a customized version of the axial score described in^21^ to integrate axial changes in the face and freezing of gait more comprehensively. While LuxPARK employed the MDS-UPDRS, the Erlangen data assessed PD symptoms of patients with the original UPDRS^22^. Differences are described in the MDS-UPDRS publication^7^. For the sake of simplicity, we refer to both, the MDS-UPDRS, as well as the original UPDRS, as UPDRS. The Erlangen data evaluated less clinical scores and thus, only UPDRS III and the axial score (without freezing of gait, as this was not part of the UPDRS, but MDS-UPDRS only) were used as outcomes. Medication status (ON/OFF) was based on physician’s judgement during the visit as reported by the corresponding UPDRS III item.

For both datasets, patients were selected, such that each patient had data from at least two visits for each outcome. This resulted in 612 PD patients from the LuxPARK cohort and 264 PD patients from the Erlangen cohort. Characteristics of these patients can be found in detail in Supplementary Table S1.

In both cohorts written informed consent was obtained, and ethical guidelines were adhered to. For LuxPARK cohort all experimental protocols were approved by the National Ethics Board in Luxembourg (CNER Ref: 201,407/13) and all methods were carried out in accordance with relevant guidelines and regulations. For Erlangen cohort all experimental protocols were approved under (IRB-approval-Re. -No. 4208, 21.04.2010, IRB, Medical Faculty, Friedrich-Alexander University Erlangen-Nürnberg, Germany) and by local ethics committee (reference number: 166_18 B, Medical Faculty, FAU Erlangen-Nürnberg, Germany). All methods were carried out in accordance with relevant guidelines and regulations.

### Gait measurements

Additional to the traditional clinical outcome scores described above, gait measurements using Portabiles digital gait device were available in both, LuxPARK and Erlangen. While in LuxPARK only one digital gait assessment, at a single visit, was performed, multiple digital gait assessments over time were available for the Erlangen patients (2.84 ± 2.37 visits per patient on average, total: 506 gait visits). Digital gait assessments in Erlangen were collected irregularly, similar to the clinical data (difference between first and second digital gait assessment: 503.11 ± 488.98 days, median = 364 days). Digital gait data was available for 343 (LuxPARK) and 802 (Erlangen) patients for multiple exercises such as Time-Up-and-Go (TUG)^23^, the Turn, manual TUG (Tray), and cognitive TUG (Count), as well as the 2 × 10m, and 4 × 10m walking tasks. A detailed description of the different tasks, including differences between Erlangen and LuxPARK, can be found in the Supplementary Material. All analysis regarding digital gait were conducted only on the subset of patients for which both longitudinal clinical features (as described above), as well as digital gait features were available. In total these were 161 patients from LuxPARK and 178 patients from Erlangen. Characteristics of these patients can be found in Table 1. A detailed description of the used device, the raw signal processing, and the derived features for further analysis along with a visualization of their distribution can be found in the Supplementary Material (Supplementary Table S2, Supplementary Figure S1, Supplementary Figure S2). A data catalogue visualizing all clinical, as well as digital gait features of both cohorts can be found here: https://p-data.molekulare-neurologie.uk-erlangen.de/.

**Table 1:** Demographic and clinical characteristics of patients.

	LuxPARK	Erlangen
Number of patients	161	178
Number of visits	5.32 ± 1.53	12.08 ± 9.12
Disease duration, years	4.92 ± 5.29	5.31 ± 4.57
Age, years	64.71 ± 10.08	62.39 ± 10.37
Sex		
Male	117 (73%)	110 (62%)
Female	44 (27%)	68 (38%)
Axial score	5.71 ± 3.79	5.35 ± 3.93
PIGD	0.56 ± 0.58	N/A
TD	0.5 ± 0.41	N/A
UPDRS I	9.28 ± 5.68	N/A
UPDRS II	10.32 ± 6.8	N/A
UPDRS III	29.89 ± 14.2	20.2 ± 11.89
H&Y		
0	1	1
1	19	25
1.5	11	14
2	93	35
2.5	20	18
3	12	35
4	4	11
5	1	2
N/A	–	37

Patients characteristics for LuxPARK and Erlangen datasets. Only the subset used for the gait analysis is described here. These are all patients having longitudinal clinical features plus digital gait features. Mean and standard deviations are shown for all characteristics but sex and Hoehn & Yahr stages, where absolute (and relative) values are shown. Values for disease duration, age, and clinical scores are reported at baseline. PIGD, TD, UPDRS I, and UPDRS II are not available in the Erlangen cohort.

## Modeling approaches

### Latent time joint mixed-effect model

The latent time joint mixed-effect model (LTJMM) was first published by Li et al.^18^. Its rational is to align trajectories of patients on a common latent (i.e. unobserved) disease timescale using a multivariate linear mixed effects model. This model is defined by fixed and random effects describing the deviation of each patient from a “mean” reference trajectory, both on the time axis, as well as with respect to the actual outcome. The model can thus describe piecewise linear progression of multiple clinical outcomes over time by estimating how far the timescale of each patient is shifted away from the timescale of the reference. In our case the timescale was the reported time since PD diagnosis. The inferred patient-specific time shift can be seen as an estimate of how far a particular patient is advanced in the severity of symptoms compared to the other patients in the same cohort. Additionally, the model generates an estimate of the patient-specific slope of disease symptoms, meaning that a large “random” slope indicates a higher speed of symptom progression compared to all other patients in the same cohort. Used outcomes were in our case the UPDRS I-III, TD, PIGD, and axial score, depending on the availability in the two different datasets. Additionally, we adjusted for age at diagnosis, sex and medication status (ON/OFF) as fixed effects. All longitudinal data, irrespective of gait data availability, was used. Mathematical details of the model are described in the Supplementary Material.

### Validation of LTJMM model

Distributions of Hoehn and Yahr (H&Y) stages were examined to validate the alignment of disease trajectories on a common disease timescale. This was based on the assumption that the shifts of each individuals’ trajectory should follow an order, which roughly agrees with the H&Y stages. Patients with lower H&Y stages, who are at the beginning of the development of the disease, should be shifted further to the left on the common disease time scale than those with a higher HY stage who are more advanced in their disease. Additionally, the accuracy of the LTJMM approach was checked by calculating the correlation between real and predicted outcomes at the last observed visit, similar to^24^. For that, a LTJMM was fitted on all outcomes, but the last measurements of each patient were excluded and predicted back by the LTJMM.

### Linear mixed models to evaluate gait parameters

Linear mixed models (LMMs) were used for associating digital gait features with different outcomes at the same visit. Therefore, clinical data at the same day of gait data assessment was matched with gait data. Only data from patients having both longitudinal clinical features, as well as digital gait features available were used in this analysis. Characteristics of this patient subset are shown in Table 1. Modeled outcomes are patient-specific a) latent disease time, b) clinical outcome scores, and c) progression rate. Both latent disease time and progression rate are a direct outcome of the LTJMM model described above. When modeling patient-specific latent disease time (interpreted as estimated time since disease onset), age at diagnosis and sex were used as adjustment covariates. In case of modeling clinical outcome scores or the patient-specific progression rate, we employed the latent disease time elapsed at the time of the clinical assessment as adjustment covariate to adjust for temporal differences between observed patient trajectories. As the latent disease time was calculated from models already adjusting for age at diagnosis and sex, we did not include them as additional adjustment covariate but only relied on the latent disease time alone. Furthermore, we added a patient-specific random intercept in case of the Erlangen data, as digital gait features were available at several time points, allowing the longitudinal nature of the patient’s data to define the random effects. Different sets of digital gait features were integrated as features in the model. First, all digital gait features were used jointly (**allGait**), then features were selected according to the individual gait tasks, and lastly, we fitted LMMs for each digital gait feature separately (**singleGait**). All these models were tested against a null model containing only adjustment covariates via a likelihood ratio test. Results were adjusted for multiple testing with the Benjamini & Yekutieli method^25^.

### Simulation of a randomized controlled study

A randomized controlled trial was simulated over a one-year observation period with either original or gait predicted UPDRS III as study endpoints. The gait predicted UPDRS III was estimated from 4 × 10m tests’ digital gait features using a linear mixed effect model. We assumed visits every 60 (bi-monthly), 30 (monthly) or 7 (weekly) days to assess the sample sizes needed for evaluating the efficacy of a potentially disease-modifying drug. We employed the Erlangen data for this task, as longitudinal digital gait features were required. Simulations were inspired by an ongoing trial^26^ and assumed a treatment effect on disease progression of 30%. Control and treatment groups were equally sized without different treatment dosage arms. A power of 80%, and a significance level of 0.01 was set as parameters for the outcome simulation of the disease progression difference between treatment and control group. Power and sample size was calculated using linear mixed-effects model, based on the method from Edland^27^, implemented in the R package *longpower* ^28^.

### Predictive ML models for disease progression

Random Forest^29^, XGBoost^30^ and Lasso^31^ were used to predict the LTJMM random slope from digital gait features. In Erlangen cohort, where multiple gait visits were conducted, measurements from first and first plus second gait visit were used for prediction. Multiple feature sets were tested: only sex and age, all digital gait features (**allGait**), and task-specific digital gait feature sets. Implementations of algorithms of scikit-learn^32^ and xgboost^30^ packages were used. Hyperparameter optimization was performed using a randomized search within an inner fivefold cross validation procedure. Hyperparameter spaces for the different algorithms can be found in Supplementary Table S3. Performance metrics were evaluated in an outer fivefold repeated cross validation procedure. Performance of the models including gait features was additionally tested with a Wilcoxon signed-rank sum test comparing its ${R}^{2}$ values to those from a model integrating only sex and age as predictors. The best performing model was selected based on these statistical test results.

Feature importance analysis was done using the SHAP method implemented in the shap python package^33^. SHAP analysis was performed on models that were trained and tuned on the entire dataset.

## Results

### Overview

**Fig. 1:**
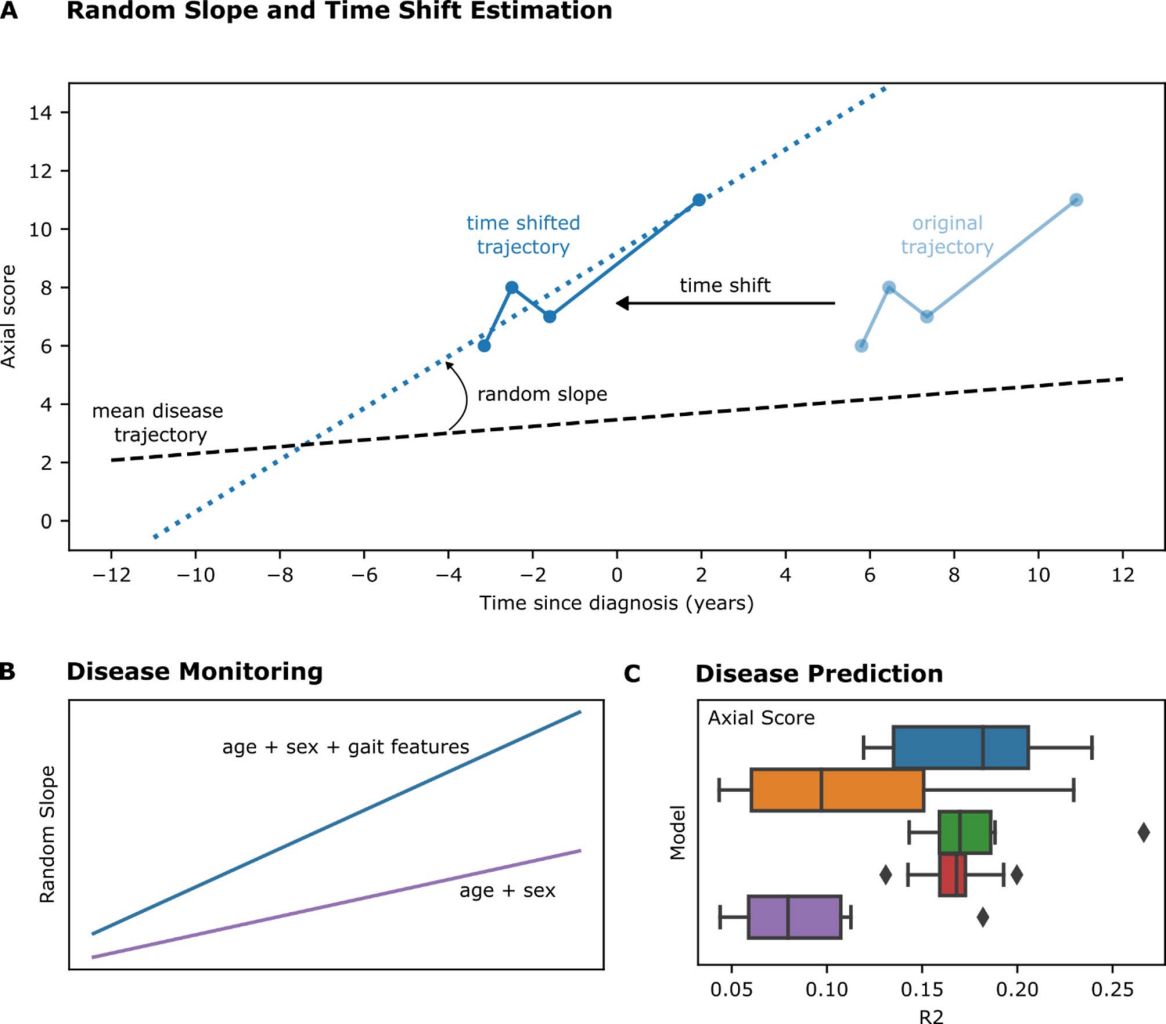
Workflow overview of digital gait analysis. Depicted is the whole workflow done in this study. (**A**) Random Slope and Time Shift Estimation: First, disease trajectories are shifted on a common disease timescale via a latent time joint mixed-effect model (LTJMM). Here, the time shift, showing the divergence from a ‘mean’ PD patient, and random slopes, a random effect measuring the progression of each patient compared to the mean patient, are calculated. (**B**) Disease Monitoring: In a next step, the variance of the random slopes is modeled with a linear (mixed) model using either only age and sex (purple) as adjustment covariates or age, sex, and digital gait features (blue). Here, multiple different sets of digital gait parameters are tested: all, task-specific, and single. Models are compared via likelihood ratio tests assessing the statistical significance of digital gait features. (**C**) Disease Prediction: Lastly, scores are predicted via a machine learning algorithm (Random Forest) with multiple digital gait feature sets (blue: all digital gait features plus age and sex, orange: TUG task plus age and sex, green: 2 × 10m task plus age and sex, red: 4 × 10m task plus age and sex, and purple: only Age and Sex).

Disease trajectories of both studies, LuxPARK and Erlangen, were first aligned on a common disease timescale via LTJMM (Fig. 1A) with the aim to adjust trajectories of each patient according to their disease status and to remove bias introduced by including patients from different stages of Parkinson’s disease. After aligning the disease trajectories on that common disease timescale, digital gait features were used as predictors in multiple settings, either for monitoring the disease severity or the disease progression (Fig. 1B) and additionally for predicting disease severity and progression (Fig. 1C).

### Latent time joint mixed-effect models for alignment of disease trajectories

LTJMM models were fitted to Erlangen and LuxPARK data. Both the original trajectories, as well as, the trajectories on the common disease timescale for each study and outcome are shown in the Supplementary Material (Supplementary Figure S3, Supplementary Figure S4). Supplementary Figure S5 and Supplementary Table S4 confirm that the model orders patient trajectories correctly according to the H&Y stages. Additionally, on average Pearson correlations of real and predicted clinical outcomes were ρ = 0.73 for Erlangen and ρ = 0.42 for LuxPARK, indicating a sufficient fit of the LTJMM models.

### Monitoring disease stage, motor symptom severity and motor symptom progression through digital gait

Statistical associations of clinical outcomes with features derived from the Portabiles digital gait device were analyzed with linear (mixed) models after aligning observed clinical outcome trajectories on a common disease timescale. We found a significant association of digital gait features with traditional clinical outcome measures in both datasets (LuxPARK: axial score, PIGD, TD and UPDRS III; Erlangen: axial score, UPDRS III) (Tables 2, 3). Clear task-specificity can be observed in LuxPark cohort, with the TUG being the most informative task. Here, the axial score was found to be highest correlated with digital gait features. When modeling the axial score with digital gait features, stance time (Count: *p* = 0.04), swing time (Tray: *p* = 0.009), gait speed (Tray: *p* = 0.029), stride length (Tray: *p* = 0.015) and the landing impact (TUG: *p* = 0.047) were found to have significant effect sizes in the models. TUG’s landing impact shows also significant effect size when modelling the PIGD score (*p* = 0.048). For the PIGD score as model outcome, we also found the gait speed (*p* = 0.014) and stride length (*p* = 0.010) significant for the Count task. Similar as in Tray task in LuxPark, we could find the swing time in Erlangen’s 4 × 10m task significant (*p* = 0.045) when modelling the axial score as outcome.

**Table 2:** Associations of digital gait features with dedicated clinical scores, their slope and latent time in LuxPARK cohort.

Outcome	All Gait	Count	Tray	TUG	Turn
Clinical score					
Axial score	**0.0055**	**0.0006**	**0.0006**	**0.0001**	**0.0175**
PIGD	0.2152	**0.0068**	**0.0068**	** < 0.0001**	*0.0629*
TD	**0.0178**	0.4882	0.4882	1	1
UPDRS I	0.2717	*0.0783*	0.2384	0.2504	*0.0783*
UPDRS II	0.4154	0.1427	0.1427	0.1427	0.3601
UPDRS III	**0.0337**	0.2149	*0.0538*	**0.0476**	0.1058
Slope (progression)					
Axial score	0.2765	0.5264	*0.0534*	*0.0990*	0.4970
PIGD	0.6788	1	1	1	1
TD	0.4895	1	0.1709	0.1709	0.6128
UPDRS I	0.3035	0.8514	*0.0621*	*0.0621*	0.5288
UPDRS II	0.3864	0.9143	0.1260	*0.0686*	0.4988
UPDRS III	0.6779	1	1	1	1
Latent time (disease stage)					
Latent time	**0.001**	**0.0288**	*0.0726*	**0.0005**	**0.0023**

Adjusted *p*-values of likelihood ratio testing the association of multiple digital gait feature sets, either all measured digital gait features or task-specific (Count, Tray, TUG, Turn), with clinical outcomes, adjusted for age at diagnosis and sex. Significant (adjusted *p* < 0.05) tests are marked in bold and weakly significant (adjusted *p* < 0.1) tests in italic. Measured outcomes are the clinical scores (top), their slope (i.e. progression, middle) and latent time (i.e. disease stage, bottom).

**Table 3:** Associations of digital gait features with dedicated clinical scores, their slope and latent time in Erlangen data.

Outcome	All Gait	TUG	2 × 10m With Stop	4 × 10m Without Stop
Clinical score				
UPDRS III	0.1574	**0.0361**	**0.0203**	**0.0110**
Axial score	**0.0010**	**0.0068**	**0.0068**	**0.0068**
Slope (progression)				
UPDRS III	1	1	1	1
Axial score	1	1	1	** < 0.0001**
Latent time (disease stage)				
Latent time	**0.0047**	** < 0.0001**	**0.0001**	** < 0.0001**

Adjusted *p*-values of likelihood ratio testing the association of multiple digital gait feature sets, either all measured digital gait features or task-specific (TUG, 2 × 10m With Stop, 4 × 10m Without Stop) with clinical outcomes, adjusted for age at diagnosis and sex. Significant (adjusted *p* < 0.05) tests are marked in bold and weakly significant (adjusted *p* < 0.1) tests in italic. Measured outcomes are the clinical scores (top), their slope (i.e. progression, middle) and latent time (i.e. disease stage, bottom).

Furthermore, a significant association with the disease stage, represented via the latent time, could be observed in LuxPARK, as well as in Erlangen (Tables 2, 3). While we could find the maximal sensor lift significant in LuxPARK for both the TUG (*p* = 0.031) and Turn (*p* = 0.048) task, the stride time in 2 × 10m (*p* = 0.001) and TUG (*p* = 0.045) task was the most informative digital gait feature in Erlangen. Visualizations of the estimates for each of the models, also showing the significance, can be found in the Supplementary Information. The estimates of each model can be found in Supplementary Table S5.

The association with the slope of the axial score (i.e. progression) was clearly significant in the Erlangen data for the 4 × 10m test and demonstrated a weak significance in LuxPARK for the Tray and TUG test. Further weakly significant associations with digital gait included the slope of the UPDRS I (Tray, TUG tests) and the slope of the UPDRS II (TUG test).

Altogether, significant associations of digital gait features were thus identifiable with traditional clinical motor scores, their slopes as well as disease stage. Results of single digital gait features tests can be found in Supplementary Table S6, generally supporting the findings reported in this section.

### Machine learning based prediction of motor symptom progression with digital gait

**Fig. 2:**
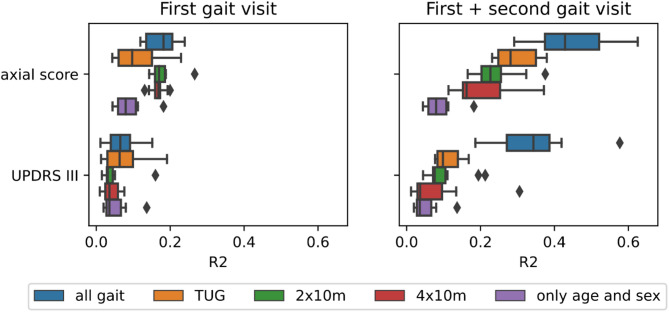
Prediction of patient-specific slopes of traditional clinical outcomes from digital gait features. Boxplots show the squared coefficient of determination (R^2^) of multiple repeats of cross-validating a Random Forest machine learning model. The model predicts the (random) slope of the axial and UPDRS III score of each patient trained on data from first gait assessment only (left) or from first plus second gait assessment (left). Different colours indicate the used feature set: all digital gait features plus age and sex (blue), TUG task plus age and sex (orange), 2 × 10m task plus age and sex (green), 4 × 10m task plus age and sex (red), and only Age and Sex (purple).

We trained different machine learning algorithms to predict the patient-specific slope (i.e. progression) of motor symptom related clinical outcomes. The strongest results could be observed via the Random Forest algorithm in a tenfold cross-validation setup (see Supplementary Figure S6 and Supplementary Figure S7) in the Erlangen data. When using only the first gait assessment in the Erlangen data for the prediction of the axial scores’ slope, a significant improvement compared to only using age and sex as predictors could be observed, specifically when using data from the 4 × 10m task (Fig. 2). Integrating also a second assessment of gait improved the prediction even more and specifically when using data from all gait tasks. Additionally, the UPDRS III slope could now be predicted significantly better compared to only using age and sex as covariates. It should be noted that similar findings could not be made in LuxPARK, because digital gait features were only available at a single visit (see Supplementary Material). Altogether, our findings reported in this section support those made in the previous section, namely that digital gait is associated with traditional motor symptom scores from the UPDRS and is sensitive to changes in those scores.

A feature importance analysis of the Random Forest models applied to the Erlangen dataset, using SHAP, revealed that gait velocity emerged as the most relevant feature. Notably, lower gait velocity, indicative of slower walking, corresponded to higher prediction values, suggesting faster progression. In addition to gait velocity, stride time, stride length, and stance time were identified as highly important features for predicting the random slope (i.e., progression) of the axial and UPDRS III scores. Specifically, increased stride and stance times, along with reduced stride lengths, were associated with faster progression. Overall, features derived from the 4 × 10m task demonstrated stronger importance than those from other tasks when incorporating all available gait features. Moreover, features from the second visit had a more pronounced impact on predictions compared to those from the first visit when both visits were considered for prediction. All SHAP plots are available in the Supplementary Material (Supplementary Figure S8, Supplementary Figure S9, Supplementary Figure S10, Supplementary Figure S11).

### Randomized controlled trial simulation shows benefit of digital gait features as endpoint

**Fig. 3:**
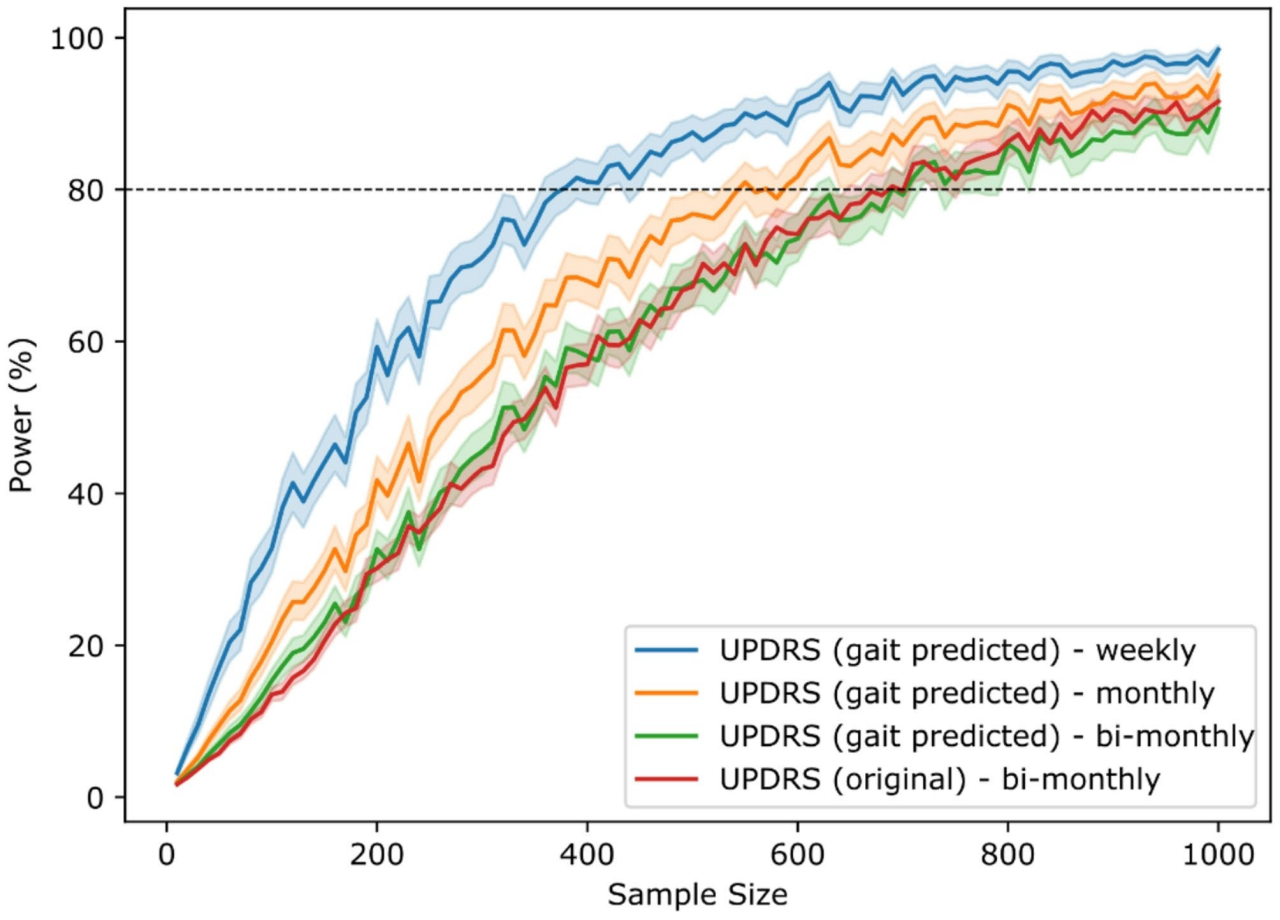
Simulation of randomized clinical trial with multiple visit frequencies. Curves describe the power a clinical study can reach with a specific sample size while expecting a treatment effect of 30% during a one year observation period and a significance level of α=0.01. Dashed line indicates 80% power. Different randomized clinical studies with either original or gait predicted UPDRS III as outcome were simulated with multiple visit frequencies. For gait predicted UPDRS III weekly (blue), monthly (orange), and bi-monthly (green) visits were simulated, while only bi-monthly visits were simulated for original UPDRS III as outcome (red)

To further evaluate the potential benefit of digital gait features compared to traditional clinical outcome scores, we simulated a longitudinal clinical trial with different visit frequencies. Notably, digital gait features can be collected more easily and cheaper at higher frequency compared to traditional clinical outcome scores, and data collection could potentially even be performed within a patient’s home environment. Estimates of the UPDRS III from digital gait features correlated well with the original data (ρ = 0.77, see Supplementary Figure S12). According to our statistical sample size calculation, which assumes a treatment effect size of 30%, bi-monthly assessments of the UPDRS III require 690 patients in total to reach 80% statistical power at a significance level of 1%. At this visit frequency the same sample size of 690 patients would be needed for a digital endpoint estimating the UPDRS III from digital gait features via the 4 × 10m task (Fig. 3). However, already a monthly assessment of this gait predicted clinical endpoint can reduce the number of patients to 550, and a weekly assessment even to 380 patients, showing a potential reduction of almost 44%. We would like to highlight once more that assessments of the traditional UPDRS III at a similar frequency in clinic would be highly challenging.

## Discussion

### Digital gait assessments for symptom monitoring and prognosis

Given the ongoing uncertainty regarding the reliability and validity of digital gait sensors in monitoring motor symptoms and their progression over time, as well as their potential benefit compared to traditional clinical scales, we conducted several analyses to evaluate the relationship between digital gait features with disease severity, disease stage, and estimated progression rates, alongside outcomes from the Unified Parkinson’s Disease Rating Scale (UPDRS).

Our findings indicate that features derived from the Portabiles digital gait device are significantly associated with disease stage (represented by latent time), disease severity assessed via the UPDRS, especially the axial score, and the progression of the axial score (represented by its slope). Importantly, the relationship between axial symptoms and digital gait features has been underexplored in the existing research literature.

### Integration of multiple gait features and gait tasks

Most studies analyzing digitally assessed gait features tend to focus on individual digital gait features rather than providing a comprehensive analysis of multiple features^8,13,16,34,35^. For instance, Kirk et al. demonstrated that gait speed is significantly lower in PD patients compared to healthy controls^8^. Similarly, Pistacchi et al. reported significant differences in stride length and step width^13^. Thus, our study addresses the limitations of these micro-level analyses by providing a comprehensive evaluation of both single and combined digital gait features. In contrast to other studies that used only one^13,16,34^ or even no standardized tasks^8^, our research required patients to perform multiple tasks, allowing us to examine task specificity and provide a systematic and unique overview of the most effective tasks for digital gait analysis. A limitation of our work in this context is that not all gait tasks were consistently performed in each study.

While combining all digital gait features proved beneficial, notable task-specific differences were observed. Interestingly, the ‘Time Up And Go’ (TUG) task consistently demonstrated effectiveness in monitoring motor symptom severity across both studies, supporting its clinical use as a standardized assessment. It was the only task able to monitor the UPDRS III in the LuxPARK cohort. Additionally, dual tasks, combining motor and cognitive exercises like the Tray and Count tasks, were associated with the PIGD score. However, we did not observe a benefit in using dual tasks compared to the traditional TUG task. Previous research has also indicated that dual tasks also can trigger Freezing of Gait^36–38^. Therefore, future research should explore the interaction between cognitive impairment and digital gait analysis.

### Predicting motor symptoms through digital gait analysis

In our study, we found a significant association between digital gait features and traditional clinical outcome measures across both datasets. Notably, clear task-specificity was observed, as discussed above. The relationship between motor symptoms and digital gait features could especially be observed for axial symptoms. This is in concordance with previous clinical studies which have shown that reduced step length and gait speed are common indicators of axial impairment in PD^39,40^. Our analysis extends these findings by confirming that digital gait analysis can reliably measure characteristics of axial impairment in PD. However, there remains a need to investigate gait asymmetry in digital gait analysis to assess its potential for improving predictions in future research. In this regard, several considerations should be addressed: 1) identifying which gait characteristics are relevant to asymmetry and 2) determining the best approach to integrate gait asymmetry into predictive models—specifically, whether to focus on the less affected or more affected side, or to evaluate the degree of asymmetry for the most insightful outcomes.

Despite this, we demonstrated that predicting the UPDRS III from digital gait data alone is a feasible task, as such estimates correlate strongly with the original data (ρ = 0.88). This finding aligns with previous studies that have successfully predicted clinical scores from digital data^41,42^. From a clinical perspective, the ability to predict the UPDRS III using only digital gait, without integrating any information about other symptoms such as hypomimia, dysarthria, or tremor, may seem surprising. However, the severity of these other symptoms may correlate with digital gait features, or they may remain relatively stable in terms of disease progression, limiting their impact on UPDRS III progression^42^. Currently, the reasons for this correlation remain unclear, and further research is needed to explore the relationship between digital gait and other PD symptoms. Additionally, the influence of the time delay to the last levodopa intake needs to be investigated, as this is not documented in our studies and thus a notable limitation of our study.

### Predicting disease stages through digital gait analysis

In our study, we found that disease stages measured through latent time could be monitored independently of the task across both cohorts. This approach contrasts with other studies distinguishing PD patients from healthy controls^5,8,13,43,44^ or classifying patients into H&Y stages based on gait data^16,34,35,45^, because these studies do not directly link digital gait features to changes in established clinical assessment scores. This is particularly relevant since the H&Y stages, commonly used to categorize PD, primarily rely on gait impairment in the higher stages^46^. As a result, distinguishing between H&Y stages through gait analysis is relatively straightforward.

In contrast, our study investigates the association between digitally assessed gait features and disease stage estimated from longitudinal trajectories of motor and non-motor symptoms, emphasizing their quantitative progression. By using the concept of latent disease time, our method offers a more gradual classification of disease progressing, including progression of multiple clinical scores. This nuanced understanding of disease progression transcends the limitations of traditional staging systems. In summary, our approach provides a more detailed and clinically applicable framework for understanding disease progression in PD and is crucial for improving the understanding and clinical acceptance of digital gait assessments.

### Predicting disease progression through digital gait analysis

There are limited studies available on the relationship between digital gait features and disease progression that indicate trends such as an increased number of steps taken or a decreased stride time over time^45,47,48^. While some research correlates clinical progression with digital gait features, only a few investigated longitudinal changes in clinical scores^47–50^. For example, Di Lazarro et al. showed that the average time to complete the TUG test is an important predictor of motor progression^47^.

A novelty of our study is that it systematically analyzes the relationship between digital gait features and disease progression, as measured by traditional clinical scales. Notably, a significant association between digital gait features and motor symptom progression derived from the UPDRS was only observed in the Erlangen dataset, where gait measurements were taken over an extended period. However, this association was only evident in the 4 × 10m walking task, underscoring the importance of longer-distance walking assessments to detect motor symptom changes in individual patients across multiple visits. This finding aligns with previous research on gait in healthy controls across different age groups, which demonstrated that at least three continuous days of at-home monitoring is necessary to detect meaningful differences in gait speed^51^.

Machine learning based prediction of the patient-level slope of UPDRS derived motor symptoms, demonstrated only a limited accuracy. When combining data from two subsequent clinical visits the coefficient of determination (${R}^{2}$) for predicting the slope of the axial score reached 0.45 and for the UPDRS III approximately 0.35. Hence, cross-sectional digital gait features alone have only limited prognostic value despite their ability to objectively monitor motor symptoms and their progression and longitudinal assessments are required for predictions of disease progression.

Feature importance analysis revealed that lower gait speed, shorter stride length, longer stride and stance time were the most important predictors of faster motor progression as measured by UPRDS III and axial score. While these gait characteristics reflect the typical PD gait pattern with shuffling, short-stepped, and slow gait, our analysis did go one step further and associate these symptoms with faster disease progression. Our results are consistent with previous studies reporting an association between digital gait markers indicating slower gait speed and faster disease progression^45,47^. Importantly, age was consistently among the top predictors of motor progression, indicating that age should always be used as a covariate in digital gait analysis to avoid bias. This is in line with the observation of reduced gait speed in healthy elderly people using digital gait analysis^51^.

When considering longitudinal data for predicting motor progression, we observed that digital gait features from the second visit were most important in predicting motor scores. This underscores that longitudinal information is most valuable for predicting disease progression which is consistent with previous research on predicting disease progression subtypes based on clinical scores using longitudinal information^24^.

Previous research has shown that patients with more asymmetric substantia nigra degeneration exhibited faster disease progression^24^. Therefore, future research should specifically investigate whether predictions of disease progression could be improved by integrating explicit information about asymmetry observed in digital gait analysis. However, such an analysis would require side-specific computations of gait features, potentially leading to less accurate gait features and a higher risk of overfitting, which in principle could also reduce model predictions.

Overall, the approach presented here demonstrates the feasibility of monitoring motor symptoms through digital gait assessment and its task specificity for individual symptoms across multiple independent studies.

### Potential value for clinical trials

Our study showed a significant association of the digital gait features with motor symptoms and disease stage. We thus performed a randomized control trial simulation, with a predicted UPDRS III outcome using the Portabiles digital gait device derived features from the 4 × 10m test. Interestingly, a digital gait predicted outcome (i.e. a digitized version of the UPDRS III), although not integrating any information about other symptoms than gait, as mentioned above, requires the same number of patients to show the efficacy of a treatment as the standard UPDRS III, if gait features are collected at the same frequency. However, a large reduction of the number of needed patients could be shown when assessing the “digital” UPDRS III via the Portabiles digital gait device in a weekly manner.

This shows the potential for using gait sensors and associated data in clinical studies, where measurements with a digital device could be taken remotely at a much higher frequency than in a traditional in-hospital setting. This may decrease the burden for patients’ quality of life and increase their willingness to contribute to a clinical study as they are not required to visit the clinic for each assessment. Additionally, data in general can be recorded via the 4 × 10m task much easier and faster than performing a complete UPDRS exam.

Only limited studies have already investigated the benefit of using digital gait device data for clinical trials. While sensor-based digital gait analysis has shown its potential to overcome limitations of UPDRS based gait assessment in a phase II trial recently^52^, most studies only refer to a subjective potential endpoint in studies, but do not test the validity of this hypothesis. Here, for the first time, an objective comparison of both outcomes, original and gait predicted UPDRS III, quantifies the potential of using sensor-based digital gait data as secondary endpoint in trials. Although the potential of the sensor-based digital gait data could clearly be seen, it does not capture quality of life aspects during a trial, which is a limitation for phase III studies and still needs to be addressed by other instruments.

## Conclusion

Overall, we have demonstrated the ability to objectively monitor the severity and progression of motor symptoms using the Portabiles HCT digital gait device. Additionally, we investigated the potential benefit of using digital gait as an outcome measure in a clinical trial context. Our work thus adds to the increasing body of literature demonstrating the benefit of digital solutions in the PD field. A distinction point is our focused digital gait device, the analysis of symptom progression and the exploration of the benefit for clinical studies.

We would like to emphasize that the use of any medical device, including the tested Portabiles digital gait device, outside pure research settings in routine medical care requires approval by a regulatory agency, and in that regard Portabiles has recently launched a request to the FDA in the USA. Besides that, it is already certified as a class I medical device (MDR) with a CE mark in Europe.

## Electronic supplementary material

Below is the link to the electronic supplementary material.

Supplementary Material 1

Supplementary Material 2

Supplementary Material 3

## Data Availability

As this study is a retrospective analysis, availability of the clinical data depends on the individual study groups. Please contact the following people for data availability: Rejko Krüger (rejko.krueger@uni.lu) for LuxPARK data and Alzhraa Ibrahim (Alzhraa.Ibrahim@uk-erlangen.de) for Erlangen data.

## Code availability

The underlying code used for training LTJMM, VaDER, and all relevant statistical analyses for this study will be published on GitHub upon acceptance of the paper under the CC BY-NC-ND 4.0 license and can be accessed via https://github.com/SCAI-BIO/PD-progression-types/.
