# Supplementary material for: Objective monitoring of motor symptom severity and their progression in Parkinson’s disease using a digital gait device
Source: Sci Rep. 2025 Jul 15;15:25541. doi: 10.1038/s41598-025-09088-7 (PMC12263828; doi:10.1038/s41598-025-09088-7)
Supplement: Supplementary file 1 — Supplementary Material 1 [file 41598_2025_9088_MOESM1_ESM.pdf]

Supplementary Information – Objective Monitoring of Motor Symptom Severity and their Progression in Parkinson's Disease Using a Digital Gait Device

Tamara Raschka[1], Jackrite To[1,2], Tom Hähnel[1,3], Stefano Sapienza[4], Alzhraa Ibrahim[5,6], Enrico Glaab[4], Heiko Gaßner[5], Ralph Steidl[7], Jürgen Winkler[5], Jean-Christophe Corvol[8], Jochen Klucken[4], Holger Fröhlich[1,2,*]

1 Department of Bioinformatics, Fraunhofer Institute for Algorithms and Scientific Computing (SCAI), Schloss Birlinghoven, 53757 Sankt Augustin, Germany

2 Bonn-Aachen International Center for IT, University of Bonn, Friedrich Hirzebruch-Allee 6, 53115 Bonn, Germany

3 Department of Neurology, University Hospital and Faculty of Medicine Carl Gustav Carus, TUD Dresden University of Technology, Dresden, Germany

4 Luxembourg Centre for Systems Biomedicine, University of Luxembourg; L-4367, Esch-sur-Alzette, Luxembourg

5 Department of Molecular Neurology, University Hospital Erlangen, Friedrich-Alexander-Universität Erlangen-Nürnberg, 91054 Erlangen, Germany

6 Machine Learning and Data Analytics Lab, Department of Artificial Intelligence in Biomedical Engineering, Friedrich-Alexander-Universität Erlangen-Nürnberg (FAU), Erlangen, Germany

7 Portabiles HealthCare Technologies, Erlangen, Germany

8 Sorbonne Université, Paris Brain Institute – ICM, Inserm, CNRS, Assistance Publique Hôpitaux de Paris, Pitié-Salpêtrière Hospital, Department of Neurology, Paris, France

* Corresponding author (holger.froehlich@scai.fraunhofer.de)

Table of contents

Methods 3
  Cohorts 3
  Gait Measurements........ 3
    Sensors 3
    Tasks 4
    Raw signal processing & Gait parameter extraction........ 4
    Derived features........ 4
    Feature distribution 5
    Latent time joint mixed-effect model........ 7
    Predictive ML models for disease progression 7
    Hyperparameter spaces 7
Results 9
  Latent-time joint mixed models for alignment of disease trajectories 9
  Monitoring disease stage, motor symptom severity and motor symptom progression through digital gait........ 12
    LuxPark 12
    Erlangen........ 13
  Machine learning based prediction of motor symptom progression with digital gait........ 14
  Randomized controlled trial simulation shows benefit of digital gait features as endpoint 21
NCER-PD/LuxPARK consortium 21
References........ 23

Methods

Cohorts

For both analyzed datasets (LuxPARK and Erlangen), patients were selected for the LTJMM modelling approach, such that each patient had data from at least two visits for each outcome. This resulted in 612 PD patients from the LuxPARK cohort and 264 PD patients from the Erlangen cohort. Characteristics of these patients can be found in **Table S1**.

	LuxPARK	Erlangen
**Number of patients**	612	264
**Number of visits**	4.46 ± 1.66	10 ± 8.52
**Disease duration, years**	4.44 ± 4.77	5.76 ± 4.93
**Age, years**	66.2 ± 10.47	62.85 ± 10.98
**Sex**		
**Male**	413 (67%)	161 (61%)
**Female**	199 (33%)	103 (39%)
**Axial score**	6.45 ± 4.62	5.66 ± 4.34
**PIGD**	0.68 ± 0.71	N/A
**TD**	0.56 ± 0.45	N/A
**UPDRS I**	10.34 ± 6.74	N/A
**UPDRS II**	11.14 ± 7.92	N/A
**UPDRS III**	33.8 ± 15.45	20.66 ± 12.63
**H&Y**		
**0**	2	1
**1**	63	36
**1.5**	45	20
**2**	322	52
**2.5**	83	25
**3**	58	47
**4**	25	25
**5**	13	3
**N/A**	1	55

Table S1: Demographic and clinical characteristics

Patients characteristics for LuxPARK and Erlangen datasets. Mean and standard deviations are shown for all characteristics but sex and Hoehn & Yahr stages, where absolute (and relative) values are shown. Values for disease duration, age, and clinical scores are reported at baseline. PIGD, TD, UPDRS I, and UPDRS II are not available in the Erlangen cohort.

Gait Measurements

Sensors

In both cohorts, an device from Portabiles (https://www.portabiles-hct.de/en/product/), which is a certified sensor-based gait device clipped to the shoe of a patient while walking, was used for collecting digital gait data. The device is currently registered for regulatory approval at the US Food and Drug Administration (FDA). Although, the same device was used, integrated sensors are slightly different. While in the LuxPark cohort, the Shimmer 3 sensors from Shimmer Sensing, Dublin, Ireland was used, where each unit consist of a tri-axial accelerometer (range ± 8g) and a tri-axial gyroscope (range ± 1000 deg/sec). The sampling rate was 102.4Hz. Within the data collection in Erlangen, multiple sensors were used. First, the Shimmer 2R inertial sensor from Shimmer Sensing, Duplin, Ireland) where each unit consists of a tri-axial accelerometer (range ± 6g) and a tri-axial gyroscope (range ± 500 deg/sec) and the sampling rate was 102.4Hz. Additionally, the Shimmer 3 sensor, as described above, and the NilsPod from Portabiles GmbH, Erlangen, Germany, were used. The NilsPod units consist of a tri-axial accelerometer (range ± 8g) and a 3-D gyroscope (range ± 2000 deg/sec) and data was recorded at a sampling rate of 102.4Hz.

Tasks

For data collection, patients had to perform multiple tasks. In the LuxPark cohort these were four different tasks:

1. Time Up and Go (TUG): The patient starts the task seated. Followed by a command of the examiner, the patient stands up, and walks 3.5 meters, turns around, walks back, and sits down again.
2. Turn: TUG test where the patient performs a 360° turn in the middle of the 3.5m in both directions.
3. Manual TUG (Tray): The patient has to balance a tray with a glass of water while performing the Turn task. Thus, it is a motor dual task.
4. Cognitive TUG (Count): The patient needs to count backward while performing the Tray task. Thus, it is a motor plus cognitive dual task.

In Erlangen three different task had to be performed by the patient:

1. Time Up and Go (TUG): Same task as in LuxPark, except the walking distance is just 3m.
2. 4x10mPrefWithoutStop (4x10m): Patients are asked to walk 10 meters at their preferred speed, turn around, walk back 10m, turn around and repeat that once again. The task is performed without pausing between the 10m distances.
3. 2x10mPrefWithStop (2x10m): Patients are asked to walk 10 meters at their preferred speed, stop for 2-3s, then turn around and walk 10 meters back to the starting point.

Raw signal processing & Gait parameter extraction

LuxPark: Digital gait features from LuxPark cohort were extracted with the proprietary algorithm provide by the manufacturer of the device Portabiles HCT. This pipeline has been developed from the work of Hannink et al. [1] and it is composed by a cascade of a template matching and a feature extraction module. The first detects in the raw data gait related events, such as heel strike and toe off, isolating the segments linked to the different side. These segments are processed by the second part of the pipeline, which estimates stride by stride biomechanical parameters using statistical model and a pre-trained convolutional neural network.

Erlangen: To get stride-wise gait parameters, individual strides were initially segmented using subsequence dynamic time warping proposed by Barth et al. [2]. Subsequently, gait events, including heel-strike, mid-stance, and toe-off, were detected using Rampp et al. [3] method. Finally, based on the detected events, different temporal and spatial parameters were calculated for each stride and the mean value of each parameter was calculated over each walking test. Gaitmap python package, encompassing this entire pipeline, was used for gait parameter extraction [4].

Derived features

Multiple gait features were derived from the raw signals. These features are:

- Swing time: Duration from Toe Off (TO) until next Heel Strike (HS).
- Stance time: Duration from HS with the surface until TO.
- Stride time: Duration of one stride. Sum of swing and stance time.
- Stride length: Distance between two consecutive HS, the length of one stride.
- Gait velocity: The average walking speed calculated by dividing the stride length by the stride time.
- Max. lateral excursion: The maximum lateral deviation of the foot in the swing phase, measured from an imaginary line between the foot’s position at start and end of the swing phase.

- Max. Sensor Lift: The maximum elevation of the heel from the ground during the swing phase.
- Max. Toe Clearance: The maximum elevation of the toe from the ground during the swing phase.
- Max. Foot Clearance: The maximum elevation of the foot from the ground during the swing phase.
- Turning Angle: The angle between the direction of the last swing phase (imaginary line between foot position at the beginning and end of the swing phase) and the orientation of the foot in the next stance phase.
- Toe Off Angle: The angle between the heel and the surface at the beginning of the swing phase.
- Heel Strike Angle: The angle between the toes and the surface when the foot lands.
- Landing Impact: The maximum vertical acceleration during landing of the foot.

An overview about which features were available in which cohort and task can be found in ***Supplementary Table S2***.

Task	Feature
**Erlangen**	
TUG	Stride time [s]
	Swing time [s]
	Stance time [s]
	Stride length [m]
	Gait velocity [m/s]
	Max. lateral excursion [m]
4x10m<br>2x10m	Stride time [s]
	Swing time [s]
	Stance time [s]
	Stride length [m]
	Gait velocity [m/s]
**LuxPark**	
TUG<br>Turn<br>Tray<br>Count	Max. Sensor Lift [cm]
	Stance time [s]
	Max. Toe Clearance [cm]
	Swing time [s]
	Max. Foot Clearance [cm]
	Gait velocity [m/s]
	Turning Angle [deg]
	Toe Off Angle [deg]
	Heel Strike Angle [deg]
	Stride length [cm]
	Stance Time (total)
	Landing Impact [g]
	Max. Lateral Excursion [cm]
	Swing time (total)
	Stride time [s]

Table S2: Gait feature used in tasks of cohorts
The table shows the gait features used in each task of the cohorts. Multiple task names in the same field apply such, that the same features were derived from the raw signals.

Feature distribution

Distributions of the derived features used in the analysis can be found in ***Supplementary Figure S1*** for the LuxPark cohort and in ***Supplementary Figure S2*** for the first gait visit in Erlangen. A data catalogue visualizing all clinical, as well as, gait features of both cohorts and for all available visits, can be found here: https://p-data.molekulare-neurologie.uk-erlangen.de/.

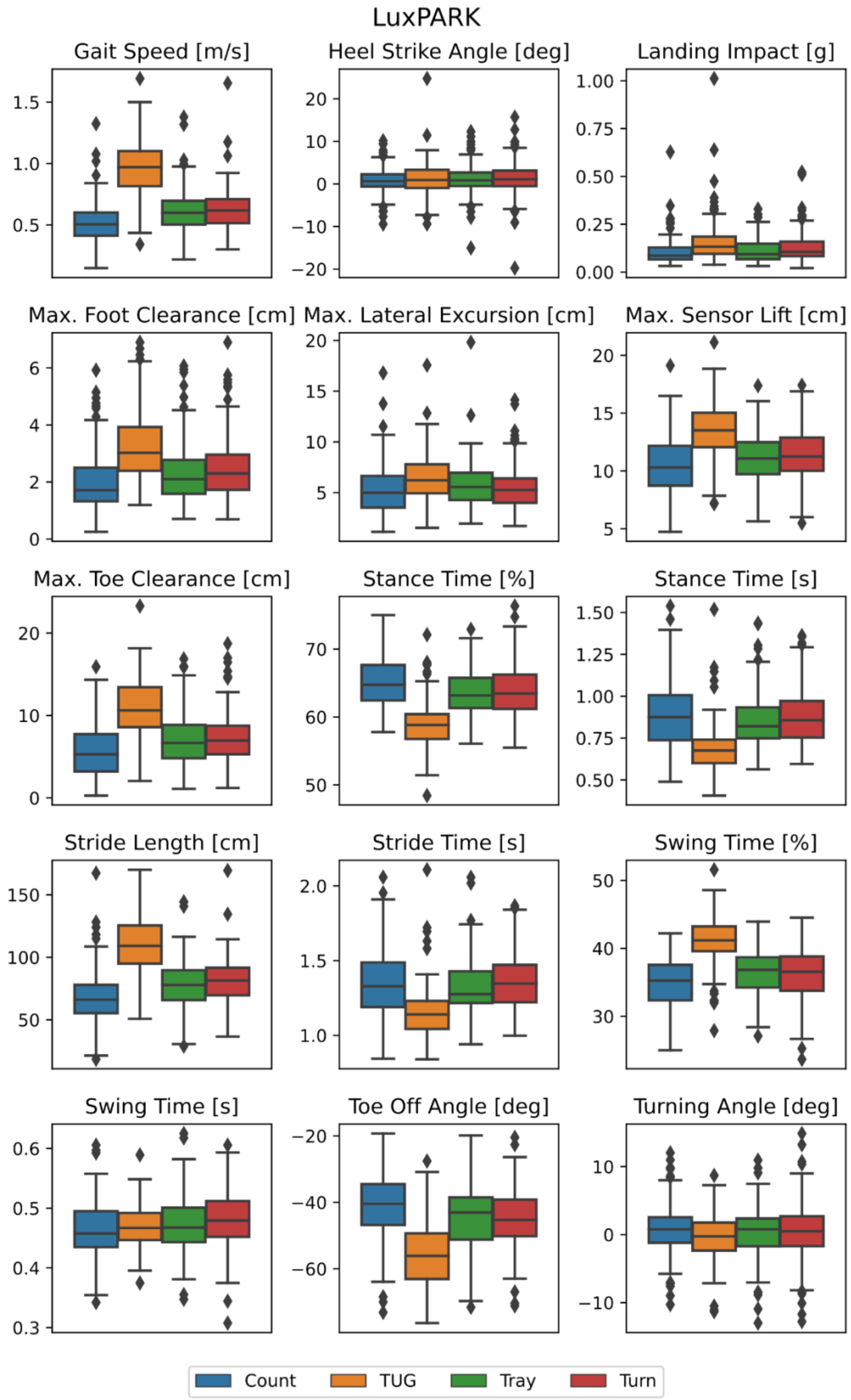

Figure S1: Gait feature distribution LuxPark
Distribution of all derived gait features in the LuxPark cohort for the four different tasks.

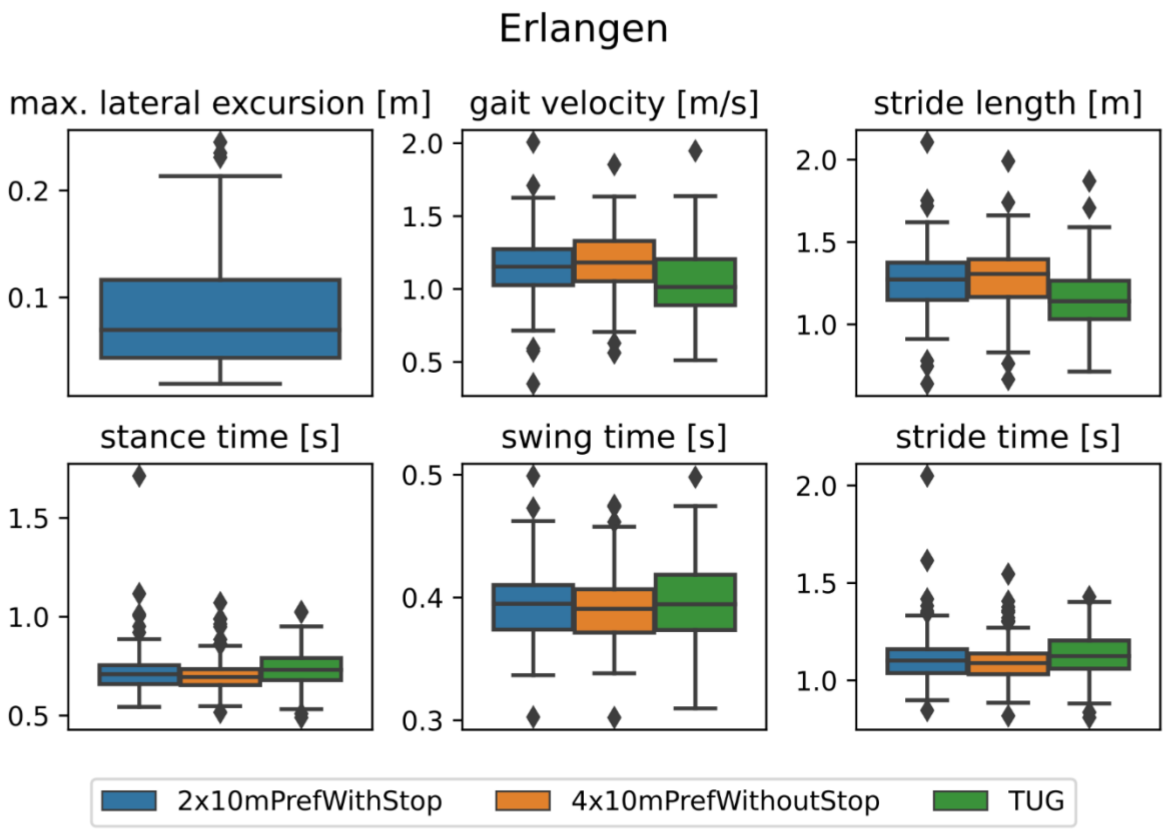

Figure S2: Gait feature distributon Erlangen
Distribution of all derived gait features om the Erlangen cohort for the three different tasks. Only data from first gait visit of each patient are shown.

Latent time joint mixed-effect model

Disease trajectories were modelled with the latent time joint mixed-effect model (LTJMM) as proposed by Li et al. [5]

$$y_{ijk} = x_i\beta_k + \gamma_k(t_{ijk} + \delta_i) + \alpha_{0ik} + \alpha_{1ik}t_{ijk} + \epsilon_{ijk}$$

Here, y_{ijk} is the modelled outcome feature *k* observed at time point *j* for an individual *i*. Age at diagnosis, sex, and medication status (ON/OFF) were included as covariates x_i in the model with β_k as corresponding coefficient shared across all individuals. The mean slope for outcome feature *k* is represented by γ_k. Here, time since diagnosis is used for modelling the time variable t_{ijk}. A per-individual timeshift is modelled with δ_i. Additionally, the model includes random effects: a random intercept α_{0ik}, as well as a random slope α_{1ik}, both modelled for each outcome and individual. Measurement error ϵ_{ijk} is drawn from a normal distribution with a zero as mean. Random effects are modelled as multivariate normal distribution with mean of zero. Implementation and analysis of LTJMM was performed using the R packages ltjmm [6] and rstan [7]. In detail, LTJMM for both LuxPARK and Erlangen cohort was performed using a Markov chain Monte Carlo (MCMC) algorithm with 4 chains, 25000 iterations and 12500 warm-up steps.

Predictive ML models for disease progression

Hyperparameter spaces

Algorithm	Parameter	Grid values
Random Forest	Max. tree depth	[10, 15, 20, 25, 30, 35, 40, 45, 50]
	Min. samples per split [%]	[0.05, 0.1, 0.15, 0.2, 0.25]

	Min. samples at each leaf node	[1, 2, 4, 8, 10, 16, 20]
	Cost-complexity pruning	[0.001, 0.01, 0.1]
XG Boost	Max. tree depth	[3, 4, 5]
	Min. child weight	[1, 5, 10]
	Gamma	[0.5, 1, 1.5, 2.5]
	Subsample	[0.6, 0.7, 0.8, 0.9, 1]
	Colsample by tree	[0.6, 0.7, 0.8, 0.9, 1]
Lasso	Alpha	[0.0001, 0.0001, 0.001, 0.01. 0.1, 0, 1, 10, 100]

Table S3: Predictive model hyperparameter space

Hyperparameter space used for hyperparameter optimization.

Results

Latent-time joint mixed models for alignment of disease trajectories

LTJMM models were fitted to Erlangen and LuxPark data. ***Supplementary Figure S3*** and ***Supplementary Figure S4*** show original and shifted disease trajectories in each cohort and for each outcome used in the study. Additionally, ***Supplementary Figure S5*** and ***Supplementary Table S4*** confirms that the model orders patient trajectories correctly according to the H&Y stages.

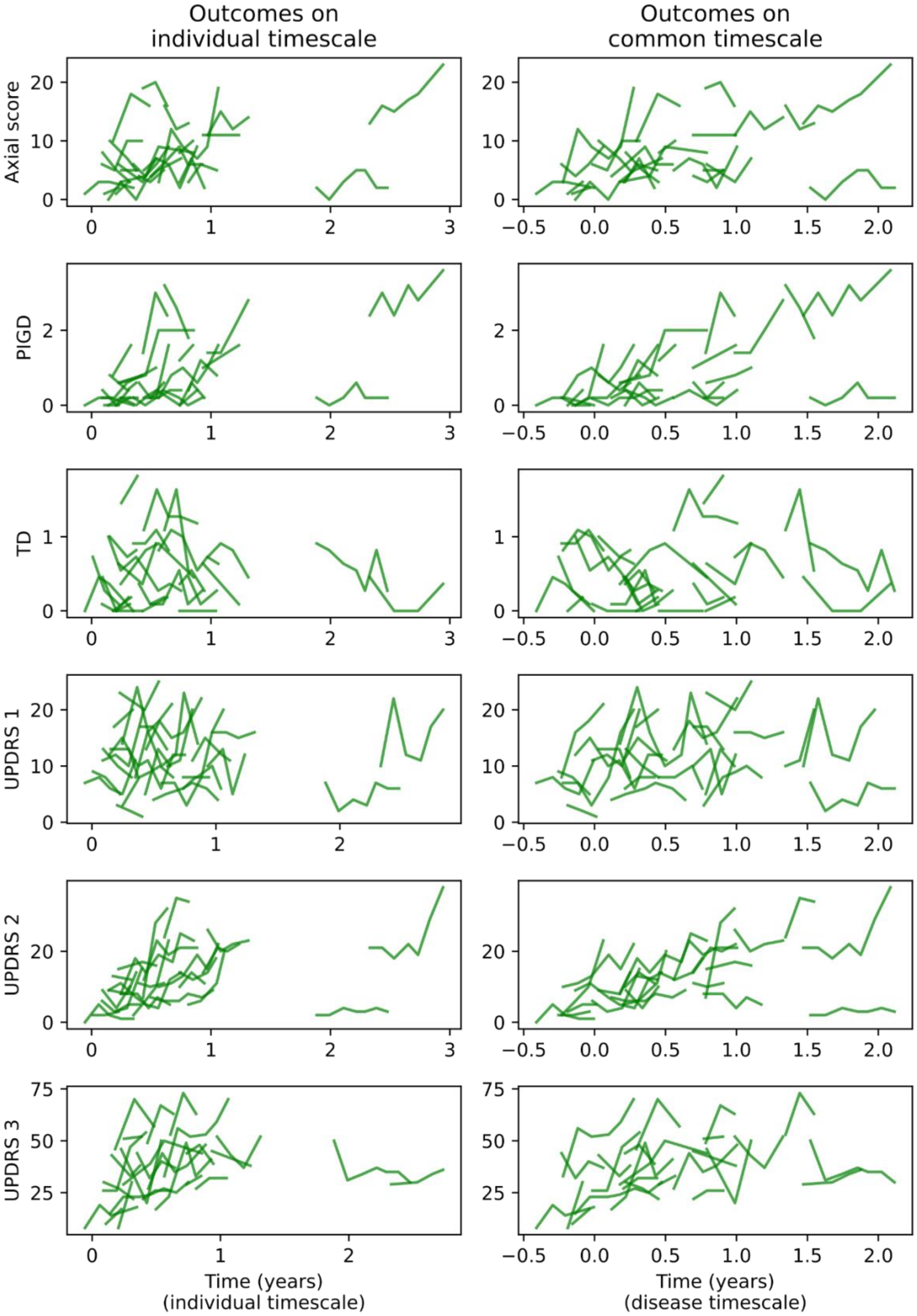

Figure S3: Original and shifted patient trajectories in LuxPARK cohort

The figures show the original (left) and shifted (right) trajectories of 20 randomly chosen patients from the LuxPARK cohort for each of the outcome used in the study.

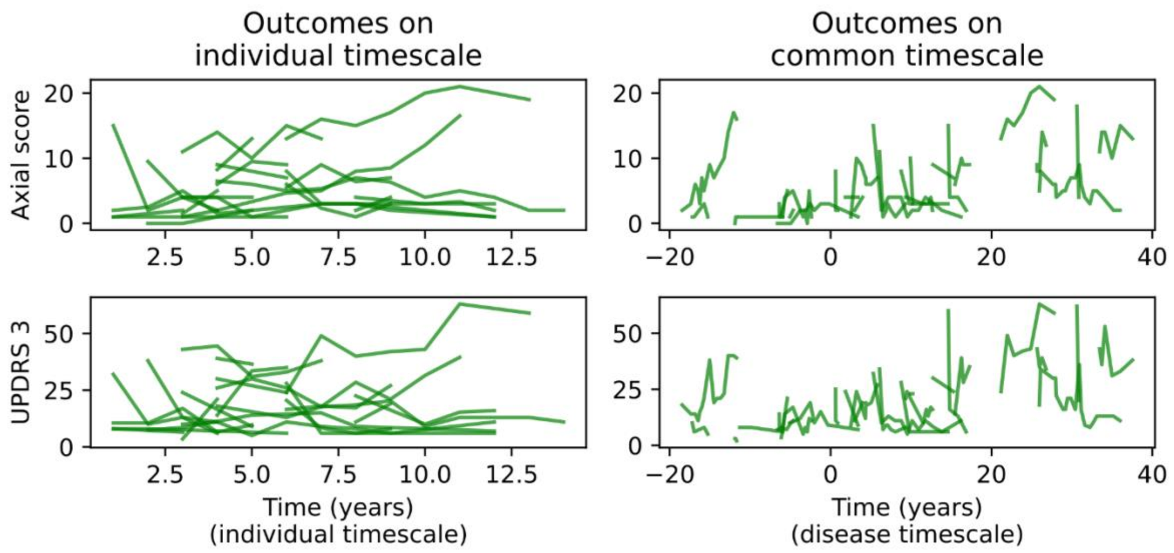

Figure S4: Original and shifted patient trajectories in Erlangen cohort
The figures show the original (left) and shifted (right) trajectories of 20 randomly chosen patients from the Erlangen cohort for each of the outcome used in the study.

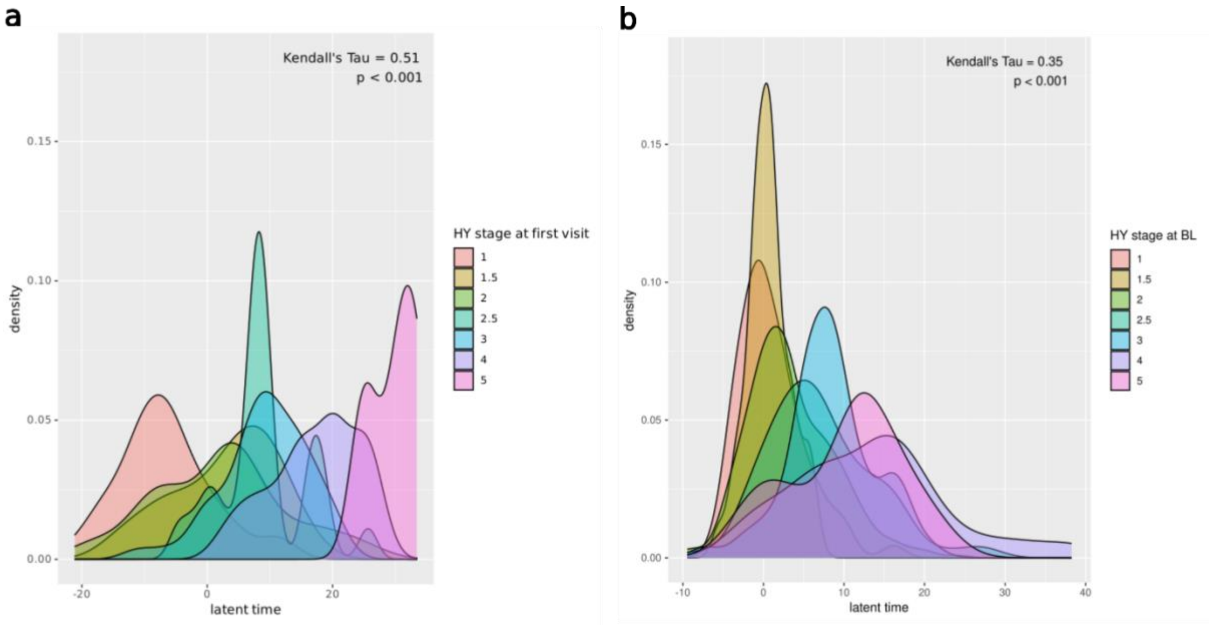

Figure S5: Latent time distributions over H&Y stages in Erlangen and LuxPark cohort
The figures show the distributions over multiple H&Y stages in a) Erlangen and b) LuxPARK cohort, confirming that the models order patient trajectories according to the disease stages. Correlation between the latent time and H&Y stages is given in terms of Kendall's tau and p-value.

H&Y stage	Erlangen		LuxPARK	
	Mean	Variance	Mean	Variance
1	-6.64	55.43	1.03	17.30
1.5	2.86	66.10	0.64	6.90
2	2.09	109.59	3.38	28.95
2.5	8.96	48.20	7.03	43.65
3	9.25	47.14	8.30	28.32
4	17.25	48.80	13.34	94.52
5	29.74	17.67	10.13	49.52

Table S4: Mean and variance of latent time of HY stages

Monitoring disease stage, motor symptom severity and motor symptom progression through digital gait

Statistical association of clinical outcomes with features collected via gait sensors were analyzed with linear (mixed) models. Additionally, to analyzing the complete set of gait features and task-specific analysis, also independent single feature analyses were done. The results of these single gait feature analysis for both Erlangen and LuxPark cohort can be found in ***Supplementary Table S6*** (Excel File). All estimates of the models integrating digital gait features can be found in ***Supplementary Table S5*** (Excel File). These estimates of the complete set of gait features and task-specific analysis are also visualized in the following plots.

Table S5: Model estimates of the linear (mixed) models [.xlsx file]
All estimates of the linear (mixed) models of Erlangen and LuxPARK ***cohort.*** *The 'Outcome' column describes the predicted outcome while the 'predictorSet' column describes the used predictors in the model. There are multiple cases: 1) all gait features, 2) task-specific gait feature sets, and 3) single gait features. The latter columns refer to the specific predictor and values show the estimate for this specific predictor.*

Table S6: Results of the linear (mixed) models for prediction with single gait parameters [.xlsx file]
Here, results of the models using a single gait parameter for prediction of latent time, random slope, and clinical scores are shown. The 'Outcome' column describes the predicted outcome while 'Task' and 'Gait parameter' refers to the single gait parameter of a specific performed gait task that was used as a predictor. 'Adjusted p-value' is the multiple testing adjusted p-value. For that the Benjamini & Yekutieli method was used.

LuxPark

Shown are always the effect sizes of each model using either all gait features as predictors or task-specific gait features.

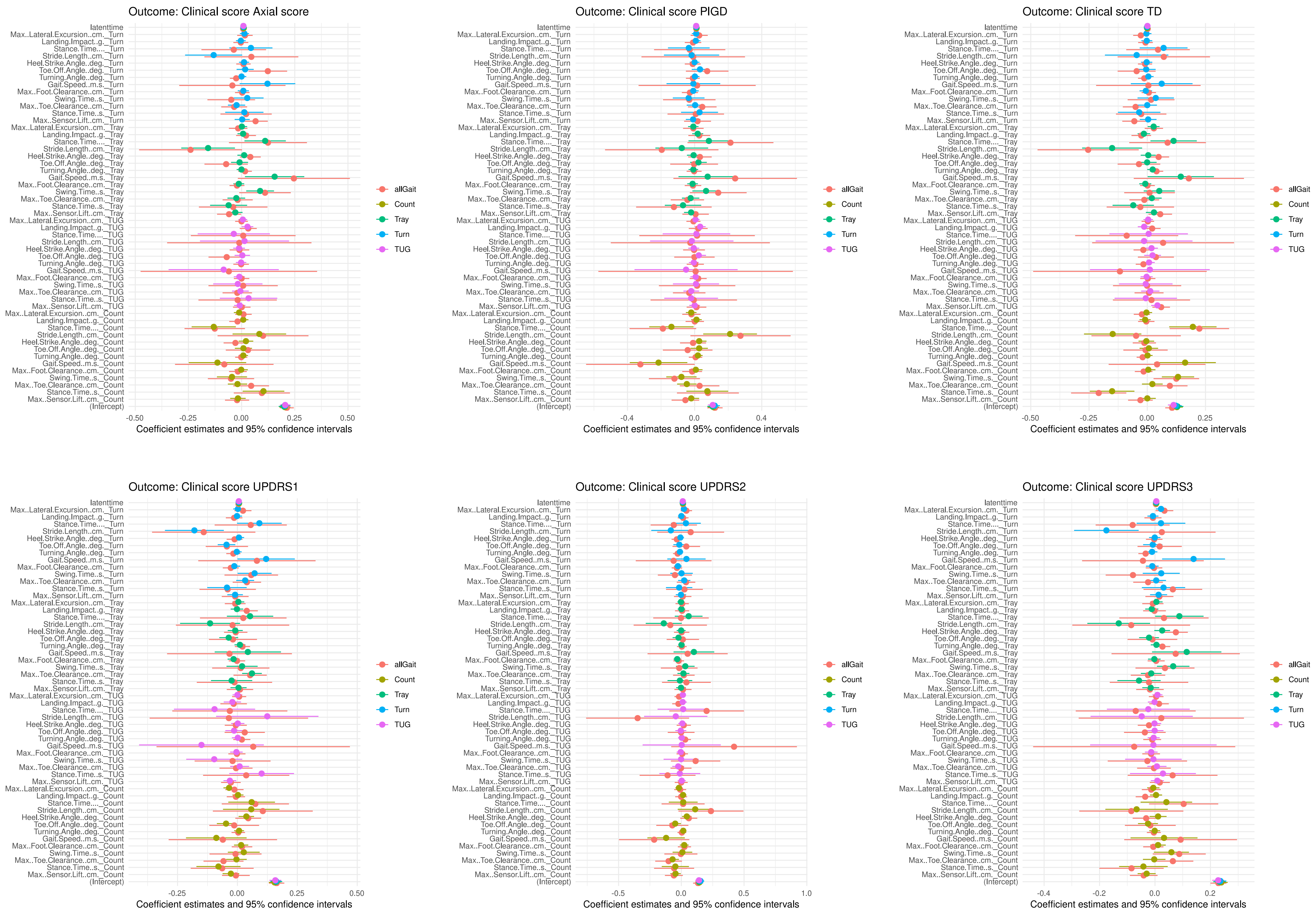

Outcome: Clinical score Axial score
Outcome: Clinical score PIGD
Outcome: Clinical score TD
Outcome: Clinical score UPDRS1
Outcome: Clinical score UPDRS2
Outcome: Clinical score UPDRS3
latenttime
(Intercept)
allGait
Count
Tray
Turn
TUG
-0.50
-0.25
0.00
0.25
0.50
-0.4
0.0
0.4
-0.5
0.5
1.0
-0.2
0.2
Coefficient estimates and 95% confidence intervals

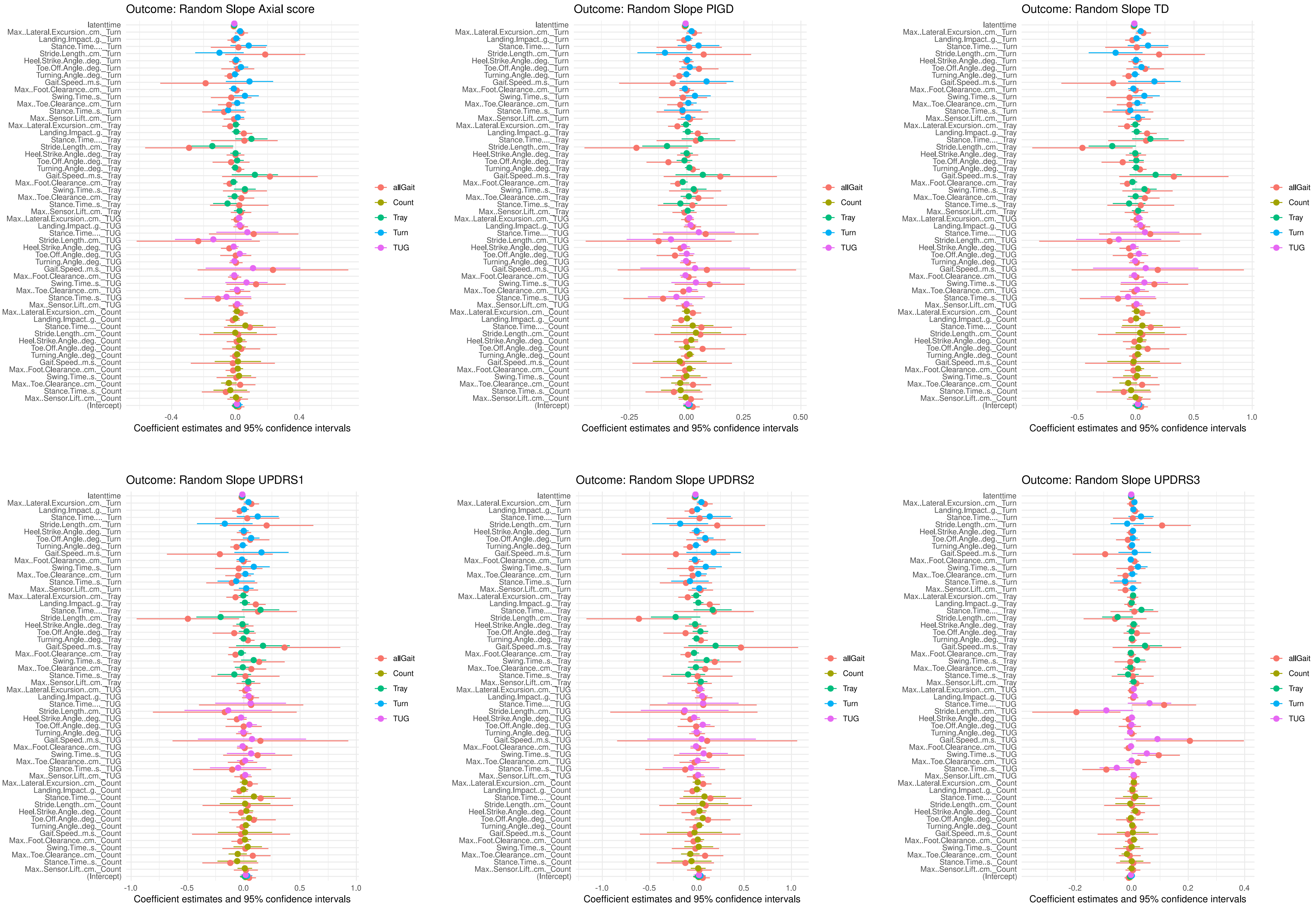

Outcome: Random Slope Axial score
Outcome: Random Slope PIGD
Outcome: Random Slope TD
Outcome: Random Slope UPDRS1
Outcome: Random Slope UPDRS2
Outcome: Random Slope UPDRS3
latenttime
Max..Lateral.Excursion..cm.._Turn
Landing.Impact..g.._Turn
Stance.Time...._Turn
Stride.Length..cm.._Turn
Heel.Strike.Angle..deg.._Turn
Toe.Off.Angle..deg.._Turn
Turning.Angle..deg.._Turn
Gait.Speed..m.s.._Turn
Max..Foot.Clearance..cm.._Turn
Swing.Time..s.._Turn
Max..Toe.Clearance..cm.._Turn
Stance.Time..s.._Turn
Max..Sensor.Lift..cm.._Turn
Max..Lateral.Excursion..cm.._Tray
Landing.Impact..g.._Tray
Stance.Time...._Tray
Stride.Length..cm.._Tray
Heel.Strike.Angle..deg.._Tray
Toe.Off.Angle..deg.._Tray
Turning.Angle..deg.._Tray
Gait.Speed..m.s.._Tray
Max..Foot.Clearance..cm.._Tray
Swing.Time..s.._Tray
Max..Toe.Clearance..cm.._Tray
Stance.Time..s.._Tray
Max..Sensor.Lift..cm.._Tray
Max..Lateral.Excursion..cm.._TUG
Landing.Impact..g.._TUG
Stance.Time...._TUG
Stride.Length..cm.._TUG
Heel.Strike.Angle..deg.._TUG
Toe.Off.Angle..deg.._TUG
Turning.Angle..deg.._TUG
Gait.Speed..m.s.._TUG
Max..Foot.Clearance..cm.._TUG
Swing.Time..s.._TUG
Max..Toe.Clearance..cm.._TUG
Stance.Time..s.._TUG
Max..Sensor.Lift..cm.._TUG
Max..Lateral.Excursion..cm.._Count
Landing.Impact..g.._Count
Stance.Time...._Count
Stride.Length..cm.._Count
Heel.Strike.Angle..deg.._Count
Toe.Off.Angle..deg.._Count
Turning.Angle..deg.._Count
Gait.Speed..m.s.._Count
Max..Foot.Clearance..cm.._Count
Swing.Time..s.._Count
Max..Toe.Clearance..cm.._Count
Stance.Time..s.._Count
Max..Sensor.Lift..cm.._Count
(Intercept)
-0.4 0.0 0.4
-0.25 0.00 0.25 0.50
-0.5 0.0 0.5 1.0
-1.0 -0.5 0.0 0.5 1.0
-1.0 -0.5 0.0 0.5 1.0
-0.2 0.0 0.2 0.4
Coefficient estimates and 95% confidence intervals
allGait
Count
Tray
Turn
TUG

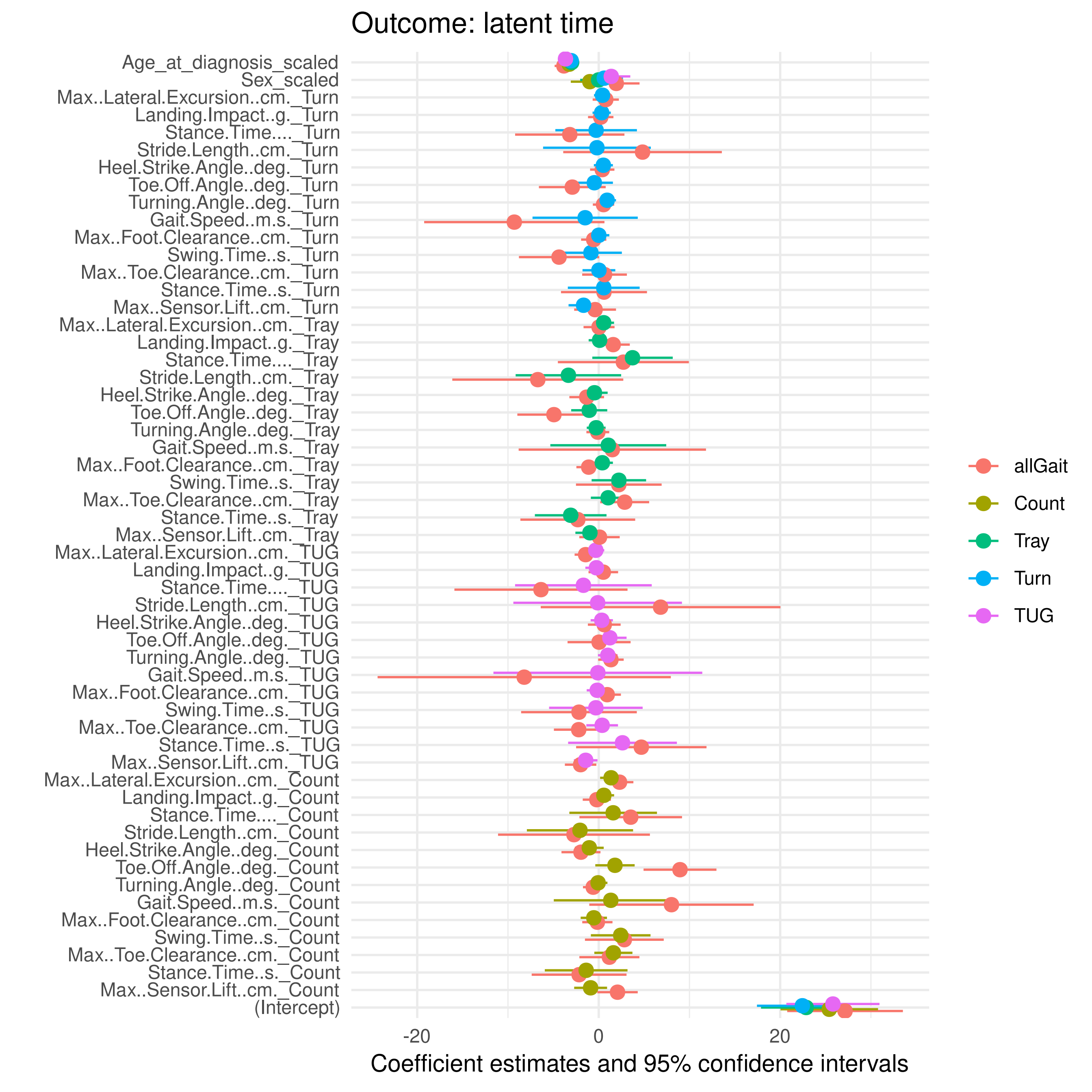

Outcome: latent time
Age_at_diagnosis_scaled
Sex_scaled
Max..Lateral.Excursion..cm._Turn
Landing.Impact..g._Turn
Stance.Time...._Turn
Stride.Length..cm._Turn
Heel.Strike.Angle..deg._Turn
Toe.Off.Angle..deg._Turn
Turning.Angle..deg._Turn
Gait.Speed..m.s._Turn
Max..Foot.Clearance..cm._Turn
Swing.Time..s._Turn
Max..Toe.Clearance..cm._Turn
Stance.Time..s._Turn
Max..Sensor.Lift..cm._Turn
Max..Lateral.Excursion..cm._Tray
Landing.Impact..g._Tray
Stance.Time...._Tray
Stride.Length..cm._Tray
Heel.Strike.Angle..deg._Tray
Toe.Off.Angle..deg._Tray
Turning.Angle..deg._Tray
Gait.Speed..m.s._Tray
Max..Foot.Clearance..cm._Tray
Swing.Time..s._Tray
Max..Toe.Clearance..cm._Tray
Stance.Time..s._Tray
Max..Sensor.Lift..cm._Tray
Max..Lateral.Excursion..cm._TUG
Landing.Impact..g._TUG
Stance.Time...._TUG
Stride.Length..cm._TUG
Heel.Strike.Angle..deg._TUG
Toe.Off.Angle..deg._TUG
Turning.Angle..deg._TUG
Gait.Speed..m.s._TUG
Max..Foot.Clearance..cm._TUG
Swing.Time..s._TUG
Max..Toe.Clearance..cm._TUG
Stance.Time..s._TUG
Max..Sensor.Lift..cm._TUG
Max..Lateral.Excursion..cm._Count
Landing.Impact..g._Count
Stance.Time...._Count
Stride.Length..cm._Count
Heel.Strike.Angle..deg._Count
Toe.Off.Angle..deg._Count
Turning.Angle..deg._Count
Gait.Speed..m.s._Count
Max..Foot.Clearance..cm._Count
Swing.Time..s._Count
Max..Toe.Clearance..cm._Count
Stance.Time..s._Count
Max..Sensor.Lift..cm._Count
(Intercept)
-20
0
20
Coefficient estimates and 95% confidence intervals
allGait
Count
Tray
Turn
TUG

Erlangen

Shown are always the effect sizes of each model using either all gait features as predictors or task-specific gait features.

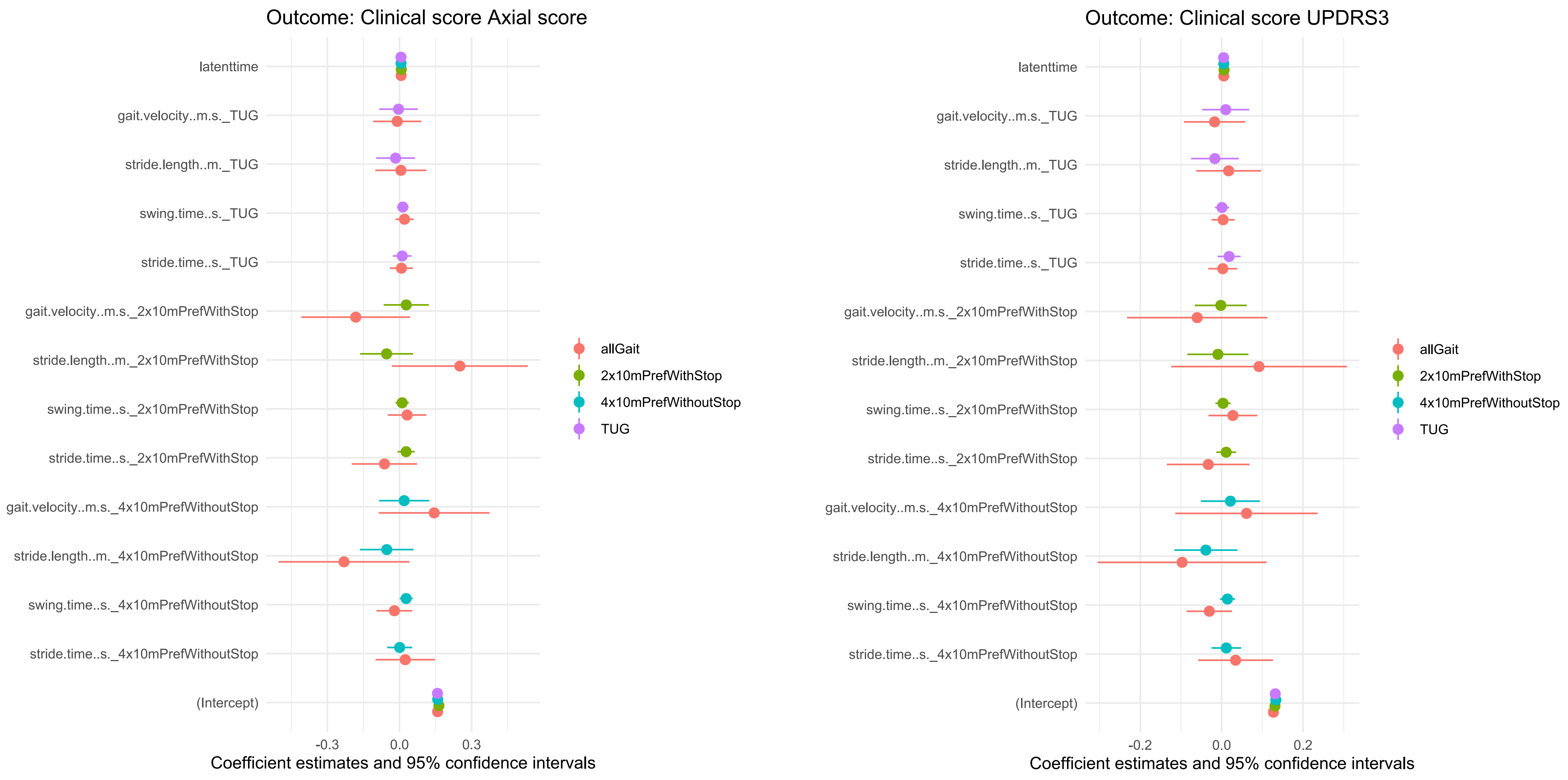

Outcome: Clinical score Axial score
latenttime
gait.velocity..m.s._TUG
stride.length..m._TUG
swing.time..s._TUG
stride.time..s._TUG
gait.velocity..m.s._2x10mPrefWithStop
stride.length..m._2x10mPrefWithStop
swing.time..s._2x10mPrefWithStop
stride.time..s._2x10mPrefWithStop
gait.velocity..m.s._4x10mPrefWithoutStop
stride.length..m._4x10mPrefWithoutStop
swing.time..s._4x10mPrefWithoutStop
stride.time..s._4x10mPrefWithoutStop
(Intercept)
-0.3
0.0
0.3
Coefficient estimates and 95% confidence intervals
allGait
2x10mPrefWithStop
4x10mPrefWithoutStop
TUG
Outcome: Clinical score UPDRS3
latenttime
gait.velocity..m.s._TUG
stride.length..m._TUG
swing.time..s._TUG
stride.time..s._TUG
gait.velocity..m.s._2x10mPrefWithStop
stride.length..m._2x10mPrefWithStop
swing.time..s._2x10mPrefWithStop
stride.time..s._2x10mPrefWithStop
gait.velocity..m.s._4x10mPrefWithoutStop
stride.length..m._4x10mPrefWithoutStop
swing.time..s._4x10mPrefWithoutStop
stride.time..s._4x10mPrefWithoutStop
(Intercept)
-0.2
0.0
0.2
Coefficient estimates and 95% confidence intervals
allGait
2x10mPrefWithStop
4x10mPrefWithoutStop
TUG

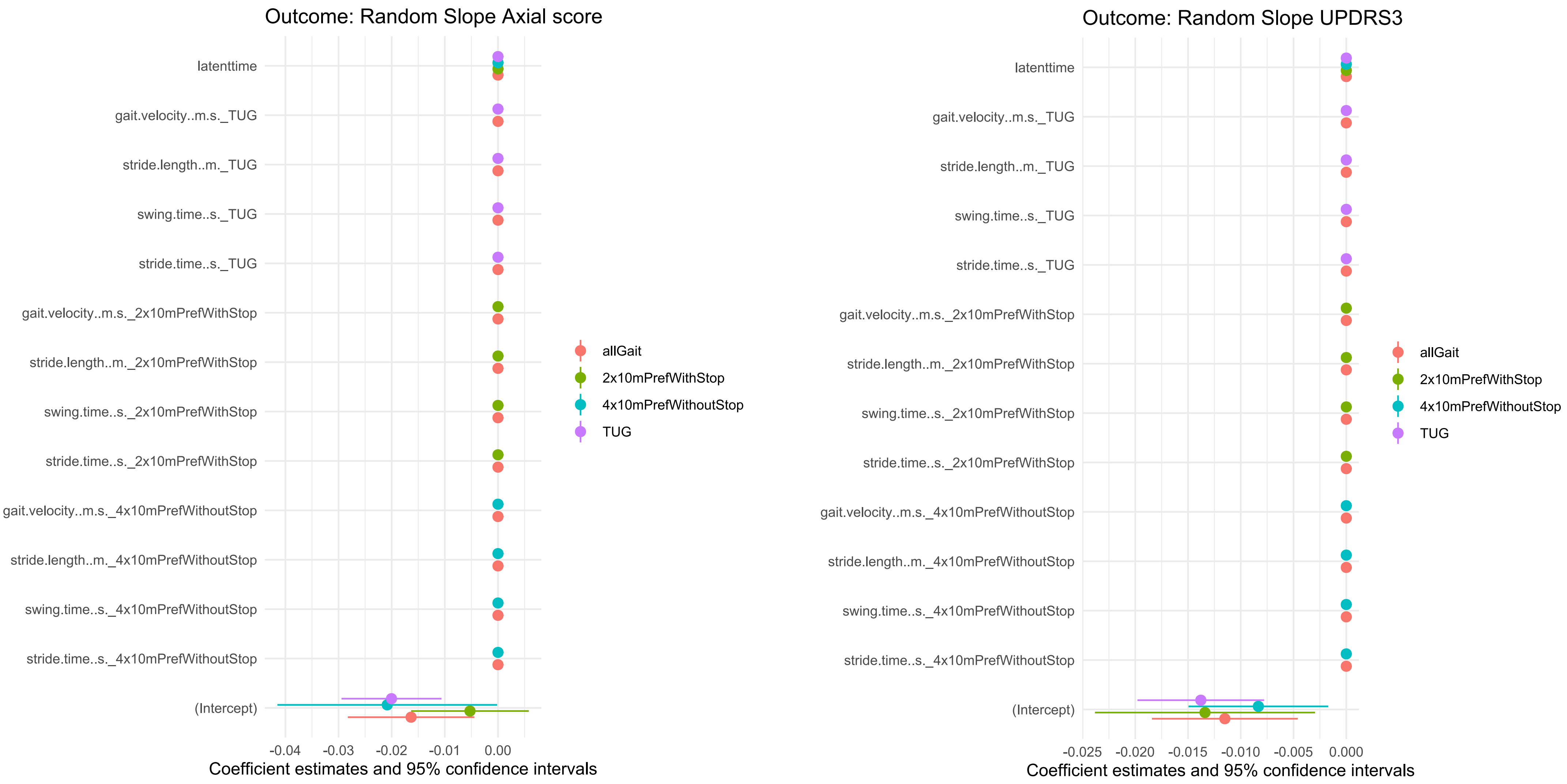

Outcome: Random Slope Axial score
latenttime
gait.velocity..m.s._TUG
stride.length..m._TUG
swing.time..s._TUG
stride.time..s._TUG
gait.velocity..m.s._2x10mPrefWithStop
stride.length..m._2x10mPrefWithStop
swing.time..s._2x10mPrefWithStop
stride.time..s._2x10mPrefWithStop
gait.velocity..m.s._4x10mPrefWithoutStop
stride.length..m._4x10mPrefWithoutStop
swing.time..s._4x10mPrefWithoutStop
stride.time..s._4x10mPrefWithoutStop
(Intercept)
-0.04
-0.03
-0.02
-0.01
0.00
Coefficient estimates and 95% confidence intervals
allGait
2x10mPrefWithStop
4x10mPrefWithoutStop
TUG
Outcome: Random Slope UPDRS3
latenttime
gait.velocity..m.s._TUG
stride.length..m._TUG
swing.time..s._TUG
stride.time..s._TUG
gait.velocity..m.s._2x10mPrefWithStop
stride.length..m._2x10mPrefWithStop
swing.time..s._2x10mPrefWithStop
stride.time..s._2x10mPrefWithStop
gait.velocity..m.s._4x10mPrefWithoutStop
stride.length..m._4x10mPrefWithoutStop
swing.time..s._4x10mPrefWithoutStop
stride.time..s._4x10mPrefWithoutStop
(Intercept)
-0.025
-0.020
-0.015
-0.010
-0.005
0.000
Coefficient estimates and 95% confidence intervals
allGait
2x10mPrefWithStop
4x10mPrefWithoutStop
TUG

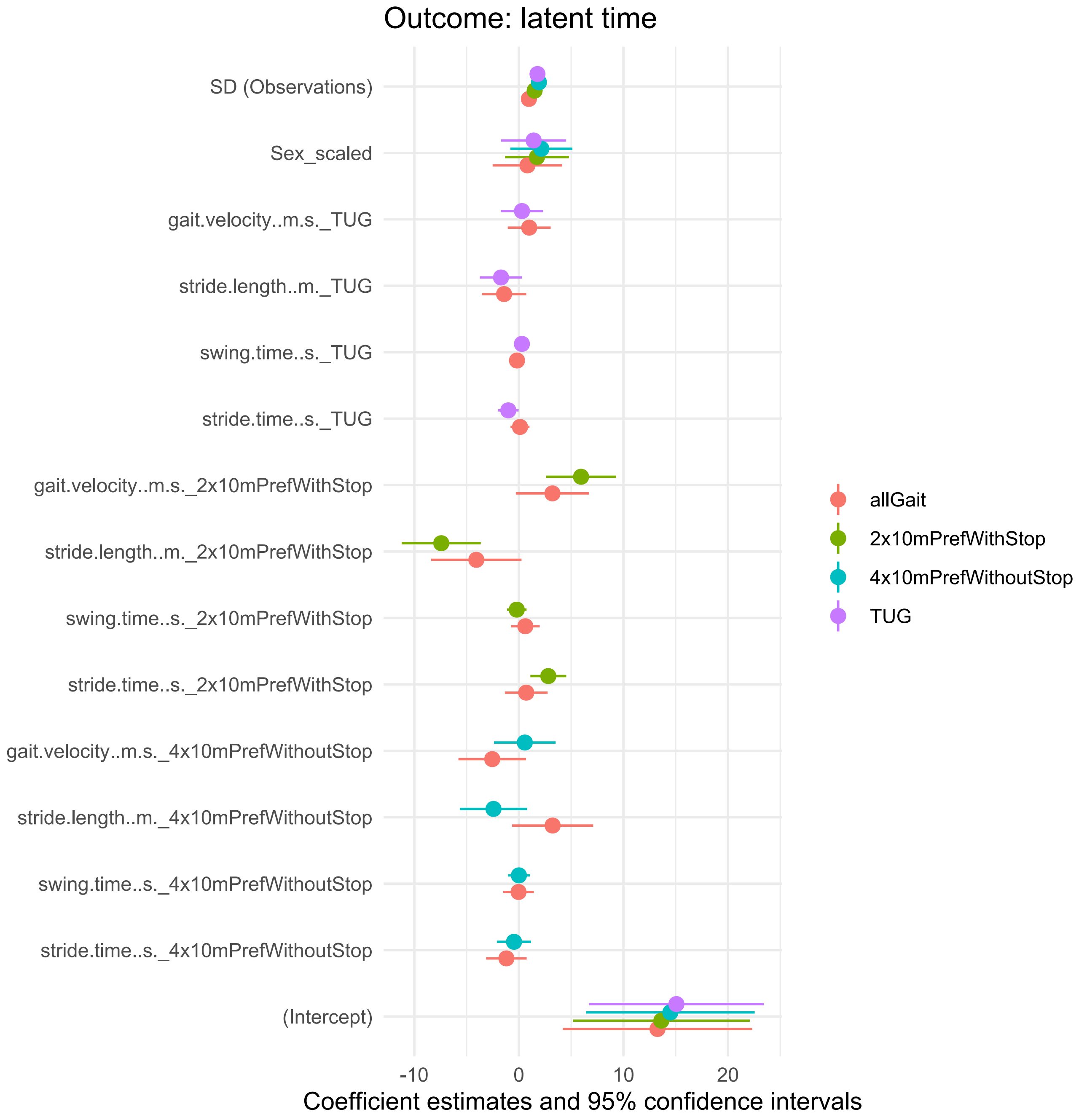
Outcome: latent time
SD (Observations)
Sex_scaled
gait.velocity..m.s._TUG
stride.length..m._TUG
swing.time..s._TUG
stride.time..s._TUG
gait.velocity..m.s._2x10mPrefWithStop
stride.length..m._2x10mPrefWithStop
swing.time..s._2x10mPrefWithStop
stride.time..s._2x10mPrefWithStop
gait.velocity..m.s._4x10mPrefWithoutStop
stride.length..m._4x10mPrefWithoutStop
swing.time..s._4x10mPrefWithoutStop
stride.time..s._4x10mPrefWithoutStop
(Intercept)
-10
0
10
20
Coefficient estimates and 95% confidence intervals
allGait
2x10mPrefWithStop
4x10mPrefWithoutStop
TUG

Machine learning based prediction of motor symptom progression with digital gait

Supplementary Figure S6 shows the results when training Random Forest, Lasso and XG Boost regression models to predict the LTJMM random slopes in the LuxPark cohort. ***Supplementary Figure S7*** shows the same type of results for the Erlangen dataset.

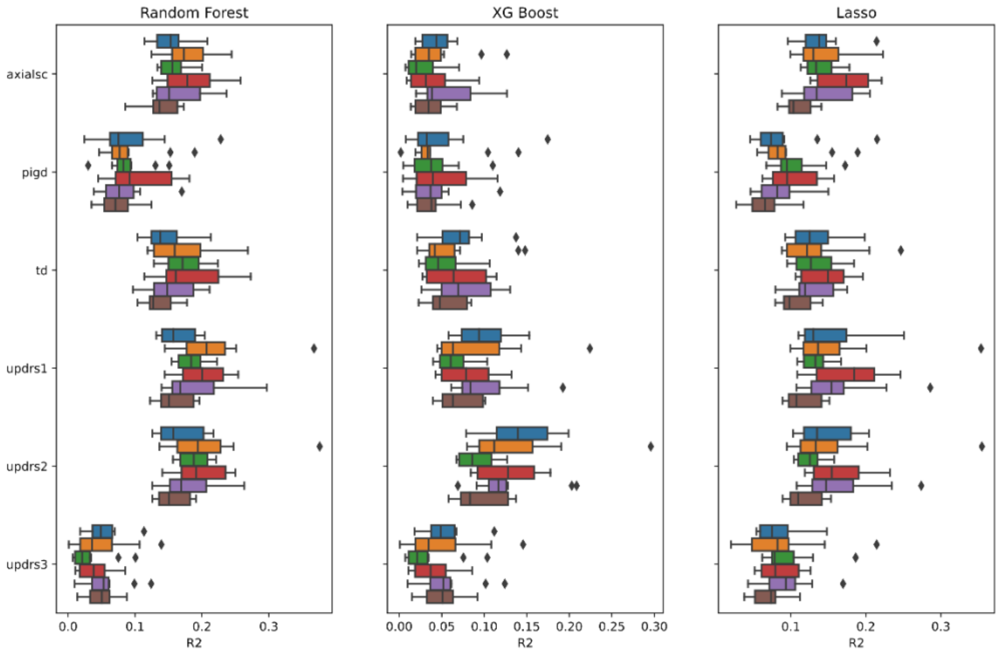

Figure S6: Cross-validation results of random slope prediction in LuxPark cohort

The boxplots show the R^2 from the repeated cross-validation for predicting multiple random slopes from either sex and age only (green), all gait features (blue), or task specific feature sets (orange: count, red: tray, purple: TUG, brown: Turn) with three different algorithms (Random Forest, XG Boost and Lasso).

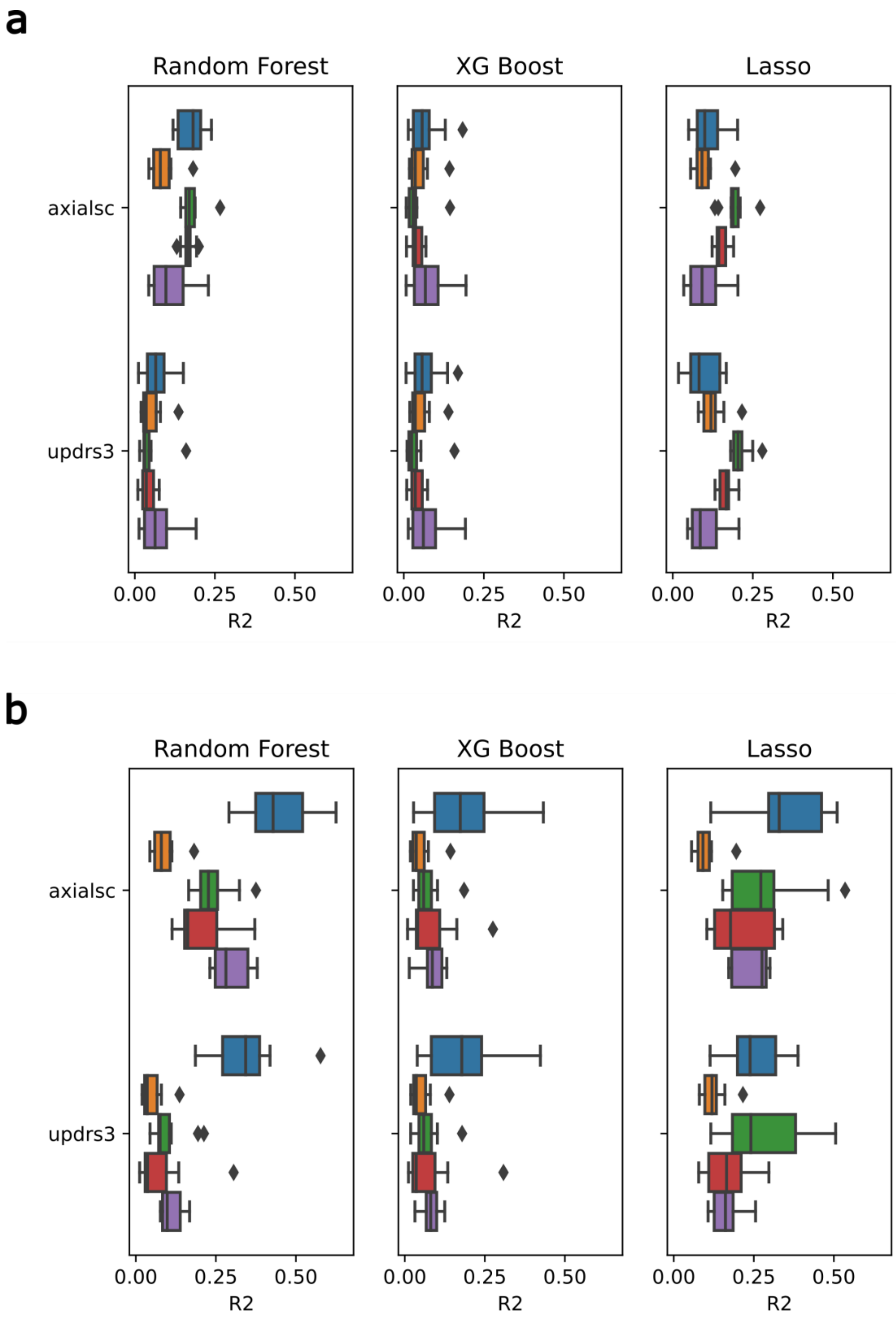

Figure S7: CV results of random slope prediction in Erlangen data

The boxplots show the R² from the repeated cross-validation for predicting multiple random slopes from either sex and age only (orange), all gait features (blue), or task specific feature sets (green: 2x10m, red: 4x10m, purple: TUG) with three different algorithms (Random Forest, XG Boost and Lasso). The plots show the results when a) only using the first gait visit and b) doing predictions based on combined first and second gait visit.

Feature Importance Analysis

Supplementary Figure S8 and ***Supplementary Figure S9*** shows the SHAP plots for the Random Forest models of the Erlangen data when predicting the random slope (i.e. progression) of the axial score and the UPDRS III score with only the first visit. ***Supplementary Figure S10*** and ***Supplementary Figure S11*** show the SHAP plots for training the models when using first and second visit data.

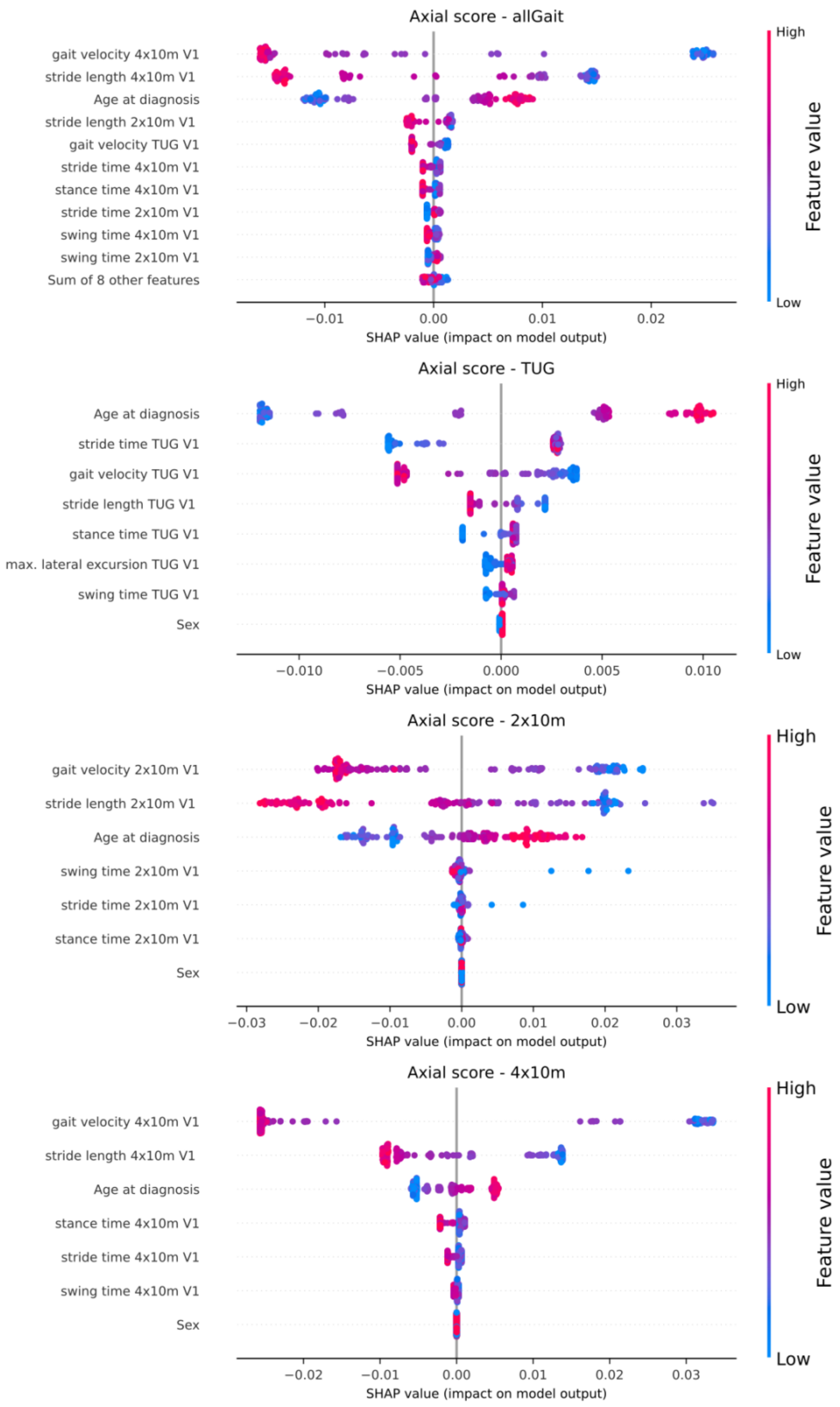

Figure S8: SHAP plot for modelling the progression of the axial score based on first visit data
SHAP values for the Random Forest model trained on the random slope of the axial score based on data from the first gait visit in the Erlangen dataset. A higher positive SHAP value indicates a higher influence of a feature to predict a patient progressing above cohort average. A more negative value indicates a higher tendency towards predicting the patient less progressing. The value of the feature is shown in a colour code, with darker red colour showing higher feature values.

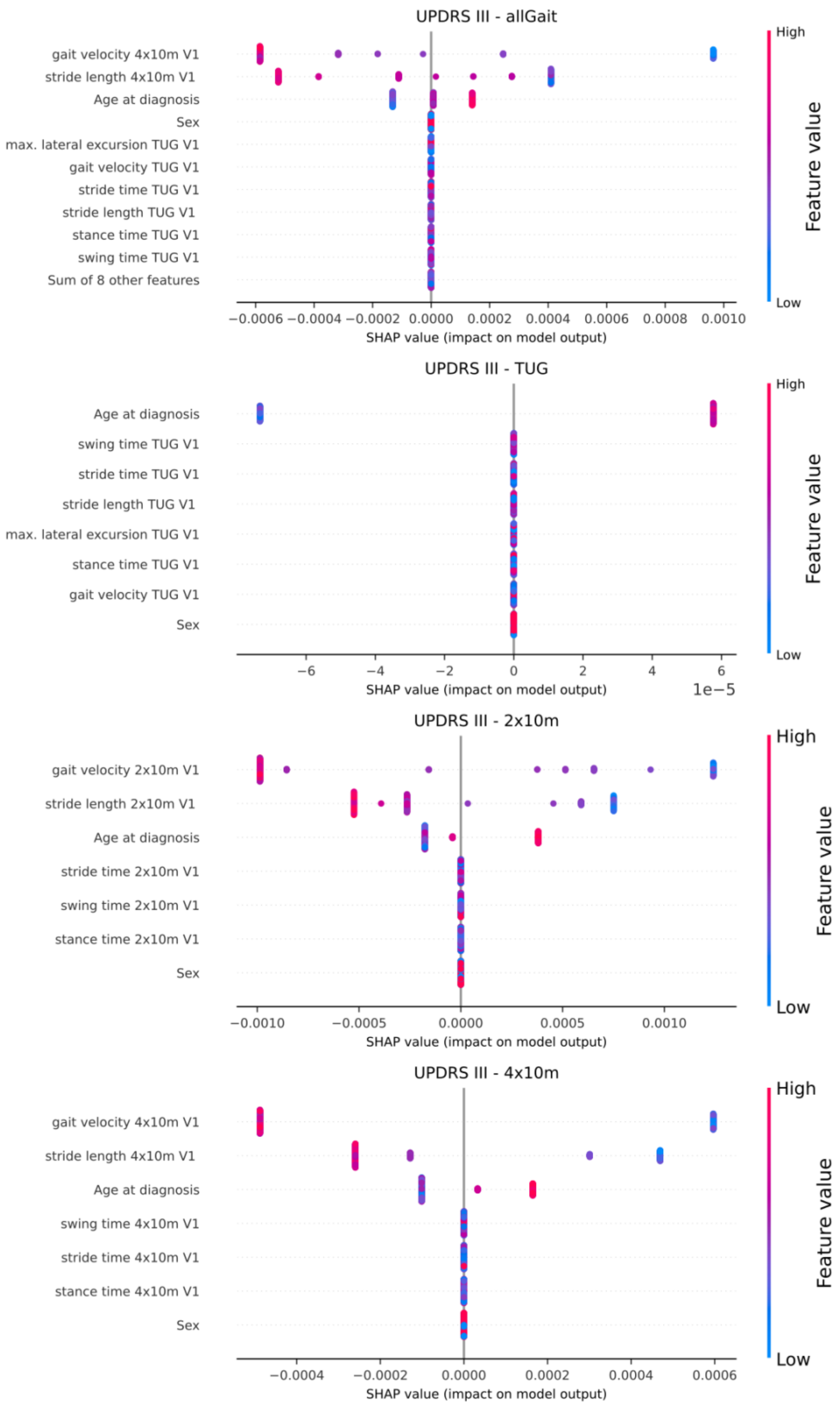

Figure S9: SHAP plot for modelling the progression of the UPDRS III score based on first visit data
SHAP values for the Random Forest model trained on the random slope of the UPDRS III score based on data from the first gait visit in the Erlangen dataset. A higher positive SHAP value indicates a higher influence of a feature to predict a patient progressing above cohort average. A more negative value indicates a higher tendency towards predicting the patient less progressing. The value of the feature is shown in a colour code, with darker red colour showing higher feature values.

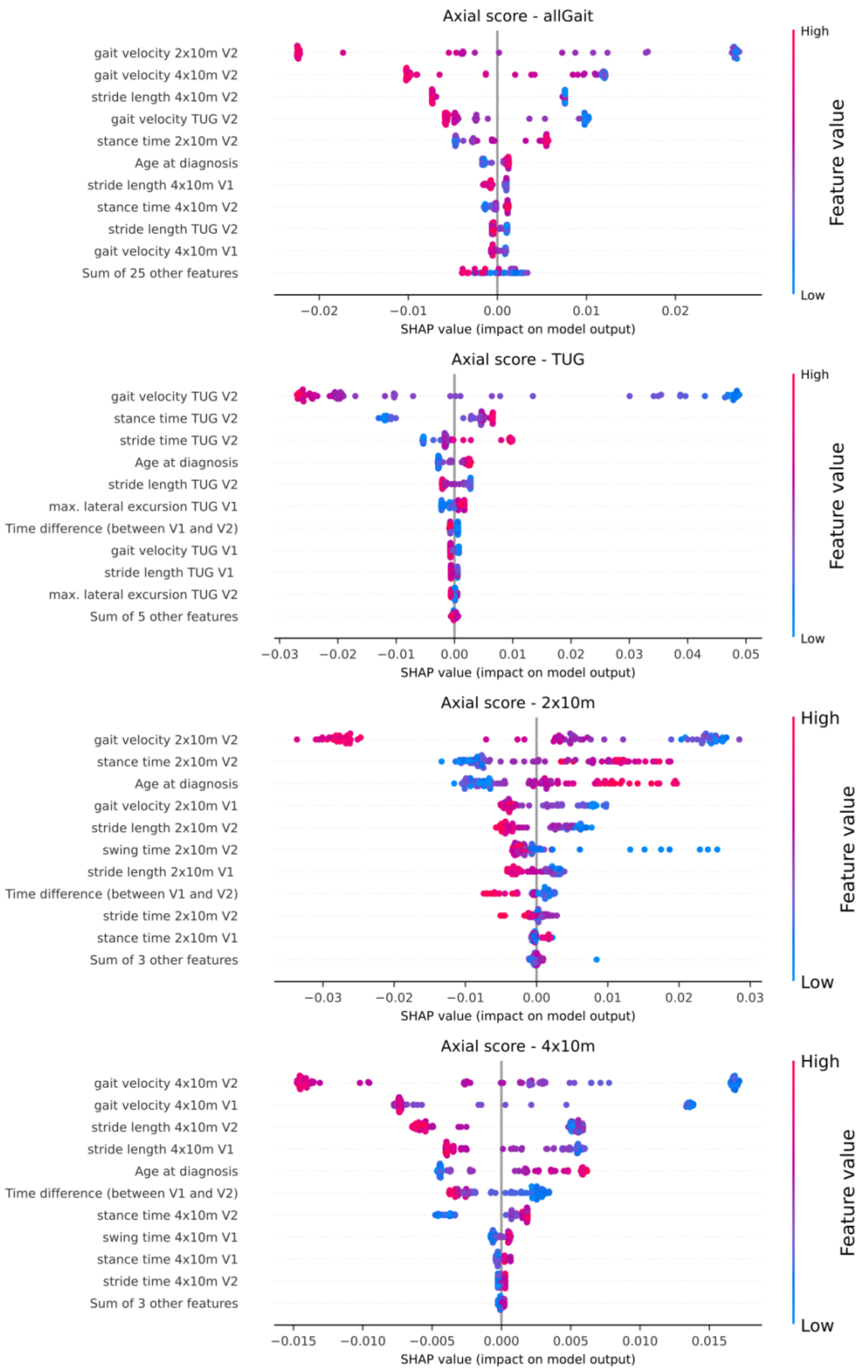

Figure S10: SHAP plot for modelling the progression of the axial score based on first and second visit data
SHAP values for the Random Forest model trained on the random slope of the axial score based on data from the first and second gait visit in the Erlangen dataset. A higher positive SHAP value indicates a higher influence of a feature to predict a patient progressing above cohort average. A more negative value indicates a higher tendency towards predicting the patient less progressing. The value of the feature is shown in a colour code, with darker red colour showing higher feature values.

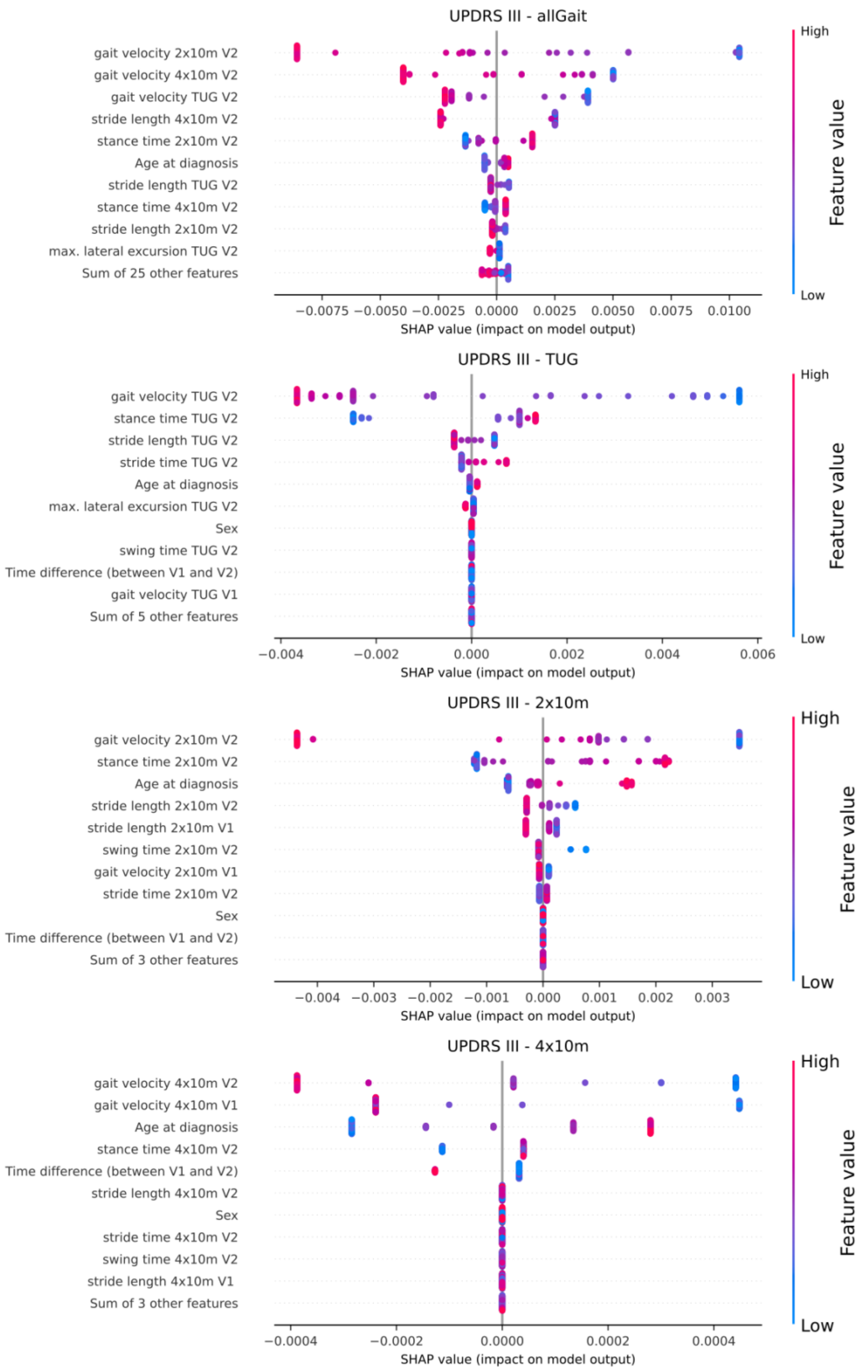

Figure S11: SHAP plot for modelling the progression of the UPDRS III score based on first and second visit data
SHAP values for the Random Forest model trained on the random slope of the UPDRS III score based on data from the first and second gait visit in the Erlangen dataset. A higher positive SHAP value indicates a higher influence of a feature to predict a patient progressing above cohort average. A more negative value indicates a higher tendency towards predicting the patient less progressing. The value of the feature is shown in a colour code, with darker red colour showing higher feature values.

Randomized controlled trial simulation shows benefit of digital gait features as endpoint

A gait predicted UPDRS III was estimated from 4x10m tests' digital gait features using a linear mixed effect model. Estimates of the UPDRS III from digital gait features correlated well with the original data (ρ=0.77). Correlation plot between original and gait predicted UPDRS III is shown in ***Supplementary Figure S12***.

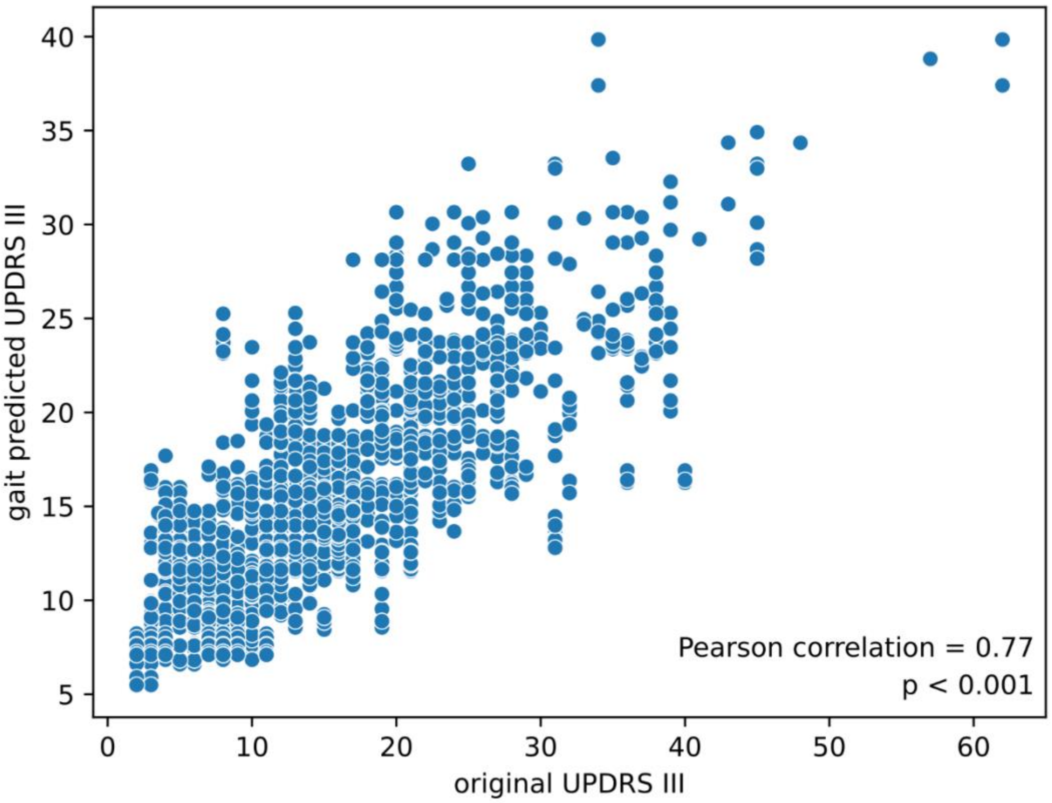

Figure S12: Scatterplot of original and gait predicted UPDRS III
The scatterplot shows the original vs. gait predicted UPDRS III, resulting in a Pearson correlation of 0.77.

NCER-PD/LuxPARK consortium

We would like to thank all participants of the Luxembourg Parkinson's Study for their important support to our research. Furthermore, we acknowledge the joint effort of the National Centre of Excellence in Research on Parkinson's Disease (NCER-PD) Consortium members from the partner institutions Luxembourg Centre for Systems Biomedicine, Luxembourg Institute of Health, Centre Hospitalier de Luxembourg, and Laboratoire National de Santé generally contributing to the Luxembourg Parkinson's Study as listed below:

Geeta ACHARYA[2], Gloria AGUAYO[2], Myriam ALEXANDRE[2], Muhammad ALI[1], Wim AMMERLANN[2], Giuseppe ARENA[1], Rudi BALLING[1], Michele BASSIS[1], Katy BEAUMONT[2], Regina BECKER[1], Camille BELLORA[2], Guy BERCHEM[3], Daniela BERG[11], Alexandre BISDORFF[5], Ibrahim BOUSSAAD[1], Kathrin BROCKMANN[11], Jessica CALMES[2], Lorieza CASTILLO[2], Gessica CONTESOTTO[2], Nico DIEDERICH[3], Rene DONDELINGER[5], Daniela ESTEVES[2], Guy FAGHERAZZI[2,] Jean-Yves FERRAND[2], Manon GANTENBEIN[2], Thomas GASSER[11], Piotr GAWRON[1], Soumyabrata GHOSH[1], Marijus GIRAITIS[2,3], Enrico GLAAB[1], Elisa GÓMEZ DE LOPE[1], Jérôme GRAAS[2], Mariella GRAZIANO[17], Valentin GROUES[1], Anne GRÜNEWALD[1], Wei GU[1], Gaël HAMMOT[2], Anne-Marie HANFF[2,20,21,] Linda HANSEN[1,3], Michael HENEKA[1], Estelle

HENRY[2], Sylvia HERBRINK[6], Sascha HERZINGER[1], Michael HEYMANN[2], Michele HU[8], Alexander HUNDT[2], Nadine JACOBY[18], Jacek JAROSLAW LEBIODA[1], Yohan JAROSZ[1], Sonja JÓNSDÓTTIR[2], Quentin KLOPFENSTEIN[1], Jochen KLUCKEN[1,2,3], Rejko KRÜGER[1,2,3], Pauline LAMBERT[2], Zied LANDOULSI[1], Roseline LENTZ[7], Inga LIEPELT[11], Robert LISZKA[14], Laura LONGHINO[3], Victoria LORENTZ[2], Paula Cristina LUPU[2], Tainá M. MARQUES[1], Clare MACKAY[10], Walter MAETZLER[15], Katrin MARCUS[13], Guilherme MARQUES[2], Patricia MARTINS CONDE[1], Patrick MAY[1], Deborah MCINTYRE[2], Chouaib MEDIOUNI[2], Francoise MEISCH[1], Myriam MENSTER[2], Maura MINELLI[2], Michel MITTELBRONN[1,4], Brit MOLLENHAUER[12], Friedrich MÜHLSCHLEGEL[4], Romain NATI[3], Ulf NEHRBASS[2], Sarah NICKELS[1], Beatrice NICOLAI[3], Jean-Paul NICOLAY[19], Fozia NOOR[2], Marek OSTASZEWSKI[1], Clarissa P. C. GOMES[1], Sinthuja PACHCHEK[1], Claire PAULY[1,3], Laure PAULY[2,20], Lukas PAVELKA[1,3], Magali PERQUIN[2], Nancy E. RAMIA[1], Rosalina RAMOS LIMA[2], Armin RAUSCHENBERGER[1], Rajesh RAWAL[1], Dheeraj REDDY BOBBILI[1], Kirsten ROOMP[1], Eduardo ROSALES[2], Isabel ROSETY[1], Estelle SANDT[2], Stefano SAPIENZA[1], Venkata SATAGOPAM[1], Margaux SCHMITT[2], Sabine SCHMITZ[1], Reinhard SCHNEIDER[1], Jens SCHWAMBORN[1], Amir SHARIFY[2], Ekaterina SOBOLEVA[1], Kate SOKOLOWSKA[2], Hermann THIEN[2], Elodie THIRY[3], Rebecca TING JIIN LOO[1], Christophe TREFOIS[1], Johanna TROUET[2], Olena TSURKALENKO[2], Michel VAILLANT[2], Mesele VALENTI[2], Gilles VAN CUTSEM[1,3], Carlos VEGA[1], Liliana VILAS BOAS[3], Maharshi VYAS[1], Richard WADE-MARTINS[9], Paul WILMES[1], Evi WOLLSCHEID-LENGELING[1], Gelani ZELIMKHANOV[3]

1. Luxembourg Centre for Systems Biomedicine, University of Luxembourg, Esch-sur-Alzette, Luxembourg
2. Luxembourg Institute of Health, Strassen, Luxembourg
3. Centre Hospitalier de Luxembourg, Strassen, Luxembourg
4. Laboratoire National de Santé, Dudelange, Luxembourg
5. Centre Hospitalier Emile Mayrisch, Esch-sur-Alzette, Luxembourg
6. Centre Hospitalier du Nord, Ettelbrück, Luxembourg
7. Parkinson Luxembourg Association, Leudelange, Luxembourg
8. Oxford Parkinson's Disease Centre, Nuffield Department of Clinical Neurosciences, University of Oxford, Oxford, UK
9. Oxford Parkinson's Disease Centre, Department of Physiology, Anatomy and Genetics, University of Oxford, South Parks Road, Oxford, UK
10. Oxford Centre for Human Brain Activity, Wellcome Centre for Integrative Neuroimaging, Department of Psychiatry, University of Oxford, Oxford, UK
11. Center of Neurology and Hertie Institute for Clinical Brain Research, Department of Neurodegenerative Diseases, University Hospital Tübingen, Germany
12. Paracelsus-Elena-Klinik, Kassel, Germany
13. Ruhr-University of Bochum, Bochum, Germany
14. Westpfalz-Klinikum GmbH, Kaiserslautern, Germany
15. Department of Neurology, University Medical Center Schleswig-Holstein, Kiel, Germany
16. Department of Neurology Philipps, University Marburg, Marburg, Germany
17. Association of Physiotherapists in Parkinson's Disease Europe, Esch-sur-Alzette, Luxembourg
18. Private practice, Ettelbruck, Luxembourg
19. Private practice, Luxembourg, Luxembourg
20. Faculty of Science, Technology and Medicine, University of Luxembourg, Esch-sur-Alzette, Luxembourg
21. Department of Epidemiology, CAPHRI School for Public Health and Primary Care, Maastricht University Medical Centre+, Maastricht, the Netherlands

References

1. Hannink J, Kautz T, Pasluosta CF, Gasmann K-G, Klucken J, Eskofier BM. Sensor-Based Gait Parameter Extraction With Deep Convolutional Neural Networks. IEEE J Biomed Health Inform. 2017;21: 85–93. doi:10.1109/JBHI.2016.2636456

2. Barth J, Oberndorfer C, Pasluosta C, Schülein S, Gassner H, Reinfelder S, et al. Stride Segmentation during Free Walk Movements Using Multi-Dimensional Subsequence Dynamic Time Warping on Inertial Sensor Data. Sensors. 2015;15: 6419–6440. doi:10.3390/s150306419

3. Rampp A, Barth J, Schuelein S, Gassmann K-G, Klucken J, Eskofier BM. Inertial Sensor-Based Stride Parameter Calculation From Gait Sequences in Geriatric Patients. IEEE Trans Biomed Eng. 2015;62: 1089–1097. doi:10.1109/TBME.2014.2368211

4. Küderle A, Ullrich M, Roth N, Ollenschläger M, Ibrahim A, Moradi H, et al. Gaitmap - An Open Ecosystem for IMU-based Human Gait Analysis and Algorithm Benchmarking. 2023 Sep. doi:10.36227/techrxiv.24047493.v2

5. Li D, Iddi S, Thompson WK, Donohue MC, Alzheimer's Disease Neuroimaging Initiative. Bayesian latent time joint mixed effect models for multicohort longitudinal data. Stat Methods Med Res. 2019;28: 835–845. doi:10.1177/0962280217737566

6. Donohue M. mdonohue / ltjmm — Bitbucket. 2017. Available: https://bitbucket.org/mdonohue/ltjmm/src/master/

7. Carpenter B, Gelman A, Hoffman MD, Lee D, Goodrich B, Betancourt M, et al. *Stan* : A Probabilistic Programming Language. J Stat Soft. 2017;76. doi:10.18637/jss.v076.i01
